# Oral presentations

**DOI:** 10.1054/bjoc.2000.1349

**Published:** 2000-08-10


British Journal of Cancer (2000) **83**(Suppl 1), 11–28
© 2000 Cancer Research Campaign
doi: 10.1054/ bjoc.2000.1349, available online at http://www.idealibrary.com on IDEAL®

### 1.1 CHARACTERISATION OF HYPOXIA RESPONSIVE ELEMENT ACTIVITY UNDER ANOXIA IN BREAST CANCER CELLS: ROLE OF HIF-1 AND ATF-4

K Ameri*[1,2], CE Lewis[1], B Burke[1] and A L Harris[2], [1]Department of Pathology University of Sheffield,[2] ICRF Medical Oncology Unit, University of Oxford, UK

Areas of hypoxia or anoxia are hallmark features of malignant tumors. Novel forms of cancer gene therapy have recently used hypoxia response elements (HREs), found in or near the promoters of oxygen-regulated genes, to target reporter or therapeutic gene expression to these low oxygen sites in solid tumors. When these cells are exposed to hypoxia, a family of hypoxia-inducible factors (HIFs) are upregulated and bind/transactivate the HRE to trigger expression of the downstream gene. It was envisaged that this would ensure transcriptional targeting only in hypoxic tumour sites and not elsewhere in the body. However, there is now concern about the degree of normoxic activity shown by such HREs as the commonly used one from the PGK-1 gene. Moreover, the activity of many HREs in complete anoxia has yet to be demonstrated. This is important as maximal HIF-1 protein levels and DNA binding occur at 0.5% oxygen in hepatoma cells, but drop markedly at 0% O2, suggesting that activity of HRE-driven DNA constructs could be compromised under anoxia (if this involves HIF-1).

In order to design HRE-gene constructs optimised to: (i) be active only in the severe hypoxia found in solid tumours, and (ii) maintain high level activity under complete anoxia in tumours, we have characterised the ability of HREs from the PGK 1, EPO and aldolase genes, to drive luciferase (LUC) expression in MCF-7 cells under normoxia (21% $O_2$), hypoxia (1 and 0.5% $O_2$) and complete anoxia (0% $O_2$). We then compared this with the activity of an anoxia-responsive VL30 element (SARE) in hypoxia/anoxia, and point mutated this to elucidate the role of the HIF-1 consensus binding site and its immediate flanking sequence in the anoxic induction of HREs in human cells. Our results show the presence of a HIF-1 consensus (CGTG) to be essential in both the anoxic as well as the hypoxic response, and that a short sequence immediately preceding this determines the magnitude of anoxia responsiveness. Immunoblotting studies revealed upregulation of HIF-1, HIF-2, ATF-1 and ATF-4 levels in 0.5 and 1%$O_2$ (compared to normoxia) in MCF-7 cells. Whereas HIF-1 and ATF-1 dropped between 0.5 and 0% $O_2$, ATF-4 levels were seen to markedly increase. Two slightly different molecular weight forms of HIF-1 were present in MCF-7 cells under 1 and 0.5%$O_2$, whereas in anoxia mainly the higher molecular weight HIF-1 was evident. We are currently investigating (i) whether the latter represents a phosphorylated form of HIF-1, and (ii) the functions of this and ATF-4 under anoxia. To date, our ATF-4 over-expression studies have indicated that ATF-4 may be involved in the transcriptional shutdown of certain promoter elements seen under anoxia, and that the SARE is protected from this by a mechanism that has yet to be defined.

### 1.2 HYPOXIA-INDUCIBLE TRANSCRIPTION FACTORS IN MACROPHAGES: IMPLICATIONS FOR OPTIMISATION OF THERAPEUTIC DNA CONSTRUCTS FOR A NOVEL, MACROPHAGE-BASED CANCER GENE THERAPY

B Burke*[1], K Ameri[1,2], N Tang[1], D Tazzyman[1], M Wells[1], A Harris[2] and C Lewis[1], [1]Dept of Pathology, University of Sheffield, Sheffield S10 2RX, [2]ICRF Medical Oncology Unit, University of Oxford, UK

The presence of areas of extremely low (hypoxia) or no (anoxia) oxygen is a hallmark feature of many types of solid human tumour. We have shown recently that macrophages accumulate in large numbers in avascular, hypoxic sites in various forms of malignant human tumour. Moreover, they upregulate a family of hypoxia-inducible transcription factors called HIFs which then bind to specific enhancers near oxygen-regulated genes, called hypoxia response elements (HREs), to trigger gene expression. These findings led to the suggestion that macrophages could be used as cellular vectors to target therapeutic genes to hypoxic tumour sites. In our work on this to date (Griffiths et al., *Gene Therapy*, 2000, 7:255), we have shown that macrophages adenovirally transduced with HRE-driven gene constructs migrate into hypoxic areas of tumour spheroids *in vitro*, where the HIFs made by the cell bind and transactivate the construct, and the therapeutic gene is expressed. This approach should ensure effective delivery to, and expression in, hypoxic tumour sites (which are relatively drug and radiotherapy resistant) *in vivo*. To date, a trimer of the HRE from the phosphoglycerate kinase-1 (PGK-1) gene has been used in these constructs, as this has been shown to be effective in primary macrophages under hypoxia. We report here on our attempts to optimise the efficacy of such hypoxia-driven constructs, and to assess a range of other HREs for efficacy, *specifically in macrophages*.

First, we have examined the effects of hypoxia on the levels of various transcription factors (HIF-1, HIF-2 (EPAS-1), Egr-1, and activating transcription factors (ATFs) 1, 2 and 4) in both the human macrophage-like cell line, Monomac 6 (MM6), and primary, monocyte-derived macrophages. Western blotting studies showed that HIF-1 and EPAS-1 proteins are induced by hypoxia in these cells, while levels of ATFs 1,2 and 4 and Egr-1 remained low or unaffected. This confirmed that HREs are currently the most appropriate response element to use for maximal hypoxia-inducibility of DNA constructs in macrophages.

We then compared the efficacy of various known HRE's (ie. derived from different hypoxia-inducible genes) in driving luciferase expression in transiently transfected (electroporated) MM6 cells under hypoxia. The highest levels of induction were observed using constructs containing either a trimer or a hexamer of the murine PGK-1 HRE. A trimer of the human erythropoietin (EPO) HRE was also hypoxiainducible in these cells. This accords well with the finding that macrophages express EPO *in vivo*. Little or no hypoxic induction was obtained using constructs carrying the anoxia/hypoxia responsive rat VL30 retrotransposon element.

### 1.3 OVER-EXPRESSION OF VASCULAR ENDOTHELIAL GROWTH FACTORS-C & -D (VEGF-C & -D) ARE ASSOCIATED WITH POOR SURVIVAL IN COLORECTAL CANCER (CRC)

JD White*, DD Kosuge, PW Hewett, TA McCulloch[1], J Carmichael & J Murray, Cancer Research Campaign Dept of Clinical Oncology & [1]Pathology Dept, Nottingham City Hospital, Nottingham, UK

**Introduction** Lymphatic involvement is important in the prognosis of colorectal cancer (CRC); however the relative impact of angiogenesis and lymphangiogenesis on prognosis of tumours is unclear. VEGF-C & -D are members of the VEGF family of angiogeneic growth factors. While VEGF-C is angiogeneic, expression also been associated with lymphatic metastasis in several cancers. VEGF-D is expressed in normal bowel mucosa and is also angiogenic, however the expression of VEGF-D in cancers is not well documented. One of the receptors for VEGF-C, VEGFR-3 is mainly expressed on lymphatic endothelial cells in the adult, thus VEGF-C may be involved in lymphatic development. We have examined the relationship between VEGF-C &-D expression and survival in CRC.

**Methods** Immunohistochemistry with polyclonal antibodies for VEGF-C &-D was performed on 84 cases of CRC and 20 adenomas. The sections were grouped according to high or low staining intensity. Associations with other prognostic features and impact on disease free (DFS) & overall survivals (OS) were assessed.

**Results** All adenomas showed low grade staining for both ligands. With progression from adenoma to advancing Dukes' stage the frequency of high-grade expression increased significantly for VEGF-C and VEGF-D. Amongst CRC samples high-grade expression was seen in 29% stained for VEGF-C and 52% for VEGF-D. High-grade expression for both ligands tended to coincide in the same sections ($p = 0.032$). Mucinous differentiation was noted in 7/8 cases with grade high VEGF-D expression. Lymphatic metastases, were seen significantly more frequently in those with high grade VEGF-D expression (48% vs 18%) corresponding figures for VEGF-C (63% vs 22%). For VEGF-C vascular invasion was seen in 6/9 high-grade compared to 16/73 low-grade tumours ($p = 0.007$). Increased expression of VEGF-D was significantly associated with increased frequency of deaths during follow up and significantly shorter DFS and OS. For VEGF-C, recurrences were significantly associated with high-grade expression, however the grade of VEGF-C expression did not predict type of recurrence. High-grade VEGF-C expression was also associated with a trend to worse OS and shorter DFS.

**Conclusions** Expression of VEGF-C &-D increases significantly with progression from adenoma to invasive carcinoma and are both associated with decreased survival. The mechanistic significance of our observations remains to be clarified.

### 1.4 TUMOUR VASCULAR TARGETING USING A NOVEL ANTI-VASCULAR ENDOTHELIAL CELL GROWTH FACTOR (VEGF) SCFV ANTIBODY

SP Cooke*, GM Boxer, DIR Spencer, L Lawrence, RB Pedley, KA Chester and RHJ Begent, Department of Oncology, RFH & UCL Medical School, Royal Free Campus, London NW3 2PF, UK

Successful antibody targeted therapy of solid tumours has been limited by a number of factors including poor penetration of therapeutic agents to all tumour cells; antigenic heterogeneity and the ability of tumour cells to become both chemo- and radio-resistant. An attractive approach is to target cytotoxic agents to the vasculature of the tumour rather than to the tumour cells themselves as restriction of one vessel is known to cause the destruction of many hundreds of tumour cells. Vascular endothelial growth factor (VEGF) is an angiogenic growth factor that is the primary stimulant of the vascularisation of solid tumours. A single-chain Fv (scFv) antibody (LL4) was selected against human $VEGF_{165}$ (VEGF-A) from a phage display library and subsequently screened by immunohistochemistry (IHC) for binding to blood vessel endothelium. Detailed analysis of the reactivity of LL4 was then carried out to determine its specificity for tumour blood vessel endothelium. This study involved further IHC screening against a panel of both normal and neoplastic human tissue sections and samples from non-tumour bearing mice. LL4 showed weak staining of some normal tissues, but strong reactivity with tumour sections demonstrating binding to VEGF in malignant cells, stroma and endothelial cell surfaces. In order to quantitate the specificity of the scFv for tumour blood vessel endothelium $^{125}$I-labelled LL4 was injected into nude mice bearing LS174T colorectal xenografts. Tumour and normal tissues were excised 1 hr and 3 hr post injection, formalin-fixed, sectioned and exposed to autoradiographic emulsion (6 weeks). Grains associated with blood vessel endothelium were counted and compared with those obtained from control mice injected with a control scFv anti-CEA antibody. Autoradiography demonstrated selective uptake and retention of LL4 in tumour xenograft compared to both the anti-CEA control ($p=0.0001$) and normal tissues ($p=0.0001$). Finally, to confirm that LL4 bound the VEGF:receptor complex, surface enhanced laser desorption ionisation affinity mass spectrometry (SELDI-AMS) was used to analyse the interaction of immobilised LL4 with recombinant VEGF-A, both free, and bound to its cognate receptor (VEGF-R2/KDR). Results showed definitively that LL4 was specific for VEGF-A both in its free state and when was complexed with its receptor. This study demonstrates specific targeting of the VEGF:receptor complex on tumour vascular endothelium by an engineered scFv antibody selected from a phage library.

This work was funded by the Cancer Research Campaign.

### 1.5 MODIFICATION OF HUMAN TUMOUR BLOOD FLOW USING PENTOXIFYLLINE, NICOTINAMIDE AND CARBOGEN

A Sibtain*[1], SA Hill[2], KA Goodchild[1], N Shah[1], MI Saunders[1], PJ Hoskin[1], [1]Marie Curie Research Wing, Mount Vemon Hospital, Northwood, HA6 2RN, [2]Gray Laboratory Cancer Research Trust, Northwood HA6 2JR, UK

**Introduction** Both nicotinamide and pentoxifylline used as single agents, when combined with carbogen breathing, result in an increase in relative red cell flux as measured by laser doppler in human tumours. Since these agents have different putative mechanisms of action, a synergistic action on blood flow might be expected.

**Aim** To assess the effect of combining oral nicotinamide, oral pentoxifylline and carbogen gas (2% $CO_2$, 98% $O_2$) breathing on human tumour blood flow. Methods and Materials: Microregional red blood cell flux was measured in accessible tumour nodules using laser Doppler microprobes in 10 patients with histologically proven malignancy. Patients received single oral doses of nicotinamide 40 mg/kg and pentoxifylline 1200 mg two hours before a ten minute period of carbogen gas breathing, corresponding to peak plasma concentrations of these drugs. Blood flow in up to six microregions in each tumour was measured for 30 minutes, recording pre-, during and post-carbogen breathing for ten minutes each.

**Results** The blood flow in 57 microregions were analysed and the mean blood flow was deduced. A mean relative increase in blood flow of 1.20 (+/–0.08, 95% CI) was observed after 8 minutes of carbogen breathing. This compares to relative increases of 1.4(+/–0.39, 95% CI) after nicotinamide alone with carbogen and 1.15(+/–0.10,95% CI) after pentoxifylline alone with carbogen. These differences are not statistically significant (p>0.05). The blood flow returned to the pre-carbogen level within 10 minutes of cessation of carbogen breathing and no patient in any group demonstrated a reduction in flow.

**Conclusion** A combination of pentoxifylline, nicotinamide and carbogen produces an increase in human tumour blood flow, similar to that observed when each of the drugs are used alone with carbogen breathing. The absence of synergism between pentoxifylline and nicotinamide suggests that despite different modes of action, a ceiling for flow enhancement may exist in tumours of around 20%.

### 1.6 ENHANCING TUMOUR VASCULARITY AND BLOOD FLOW DOES NOT INCREASE METASTASIS IN A MODEL OF COLORECTAL CANCER

P Mathur*[1], MM Davies[1], P Carnochan[2], TG Allen-Mersh[1], [1]Department of Surgery, Imperial College School of Medicine, Chelsea & Westminster Hospital, London, SW 10 9NH, [2]Institute of Cancer Research, Sutton, UK

**Background** Primary tumour vascularity correlates with risk of metastasis but it is not clear whether this is because increased vascularity improves tumour cell access to the circulation or because both tumour vascularity and metastasis are associated with the same oncogene mutations. To assess this we examined metastasis, in the same tumour cell line, where tumour vascularity was increased by either primary tumour interstitial or systemic infusion of basic Fibroblast Growth Factor (bFGF).

**Methods** Subcutaneous DHD/K12/Tr (rat colonic adenocarcinoma) tumours were infused either interstitially or systemically with bFGF. Control animals received saline. On day 28 the primary tumours were excised to allow vascularity (vessel count & vessel volume), blood flow (autoradiography) and tumour growth (tumour volume & proliferation index) assessment. The animals were recovered to allow metastasis growth and sacrificed two months later. At this stage lungs were excised, weighed and examined for metastases and extra-pulmonary metastases noted.

**Results**

| Infusion (n) | Vascularity† Count/mm² | Volume% | Tumour Volume mm³† | ProL Index %† | Rats with lung mets (n) |
|---|---|---|---|---|---|
| saline (17) | 35 (34–36) | 3.6 (2.5–4) | 1150 (1022–1277) | 12 (9.4–15) | 9 |
| interstitial bFGF (18) | 65 (62–77) | 5.9 (5.8–6) | 2268 (1882–2416) | 21 (19–23) | 11 |
| ***p**** | ***0.008*** | ***0.004*** | ***0.05*** | ***0.03*** | ***0.3*** |
| saline (13) | 10 (10–20) | 1.8 (1.3–2) | 359 (125–515) | 5 (4–6) | 7 |
| systemic bFGF (15) | 36 (31–56) | 6.4 (5.5–7) | 611 (469–628) | 14 (10–16) | 9 |
| ***p**** | ***0.009*** | ***0.009*** | ***0.0758*** | ***0.0367*** | ***0.87*** |

[*Mann Whitney-U test; † Median (IQR); ‡Repeated measures ANOVA].

There were also significant increases in vessel length density (interstitial: p=0.0001; systemic: p< 0.0001) and blood flow (interstitial: p=0.0005; systemic: p<0.0001‡) . There was no difference in lung weights (gms)(Interstitial: p=0.3; Systemic: p=0.24)*.

**Conclusion** There were significant increases in primary tumour vascularity, blood flow and growth with both interstitial and systemic bFGF infusion, however this was not associated with increased metastasis either when the primary tumour only (interstitial) or when the metastatic site (systemic) was exposed to bFGF. These results suggest that the relation between vascularity and metastasis does not result from increased tumour cell access to the circulation or increased bFGF at the metastatic site.

### 1.7 COMBRETASTATIN A4 PHOSPHATE (CA4P) TARGETS VASCULATURE IN ANIMAL AND HUMAN TUMOURS

Galbraith SM*, Taylor NJ, Maxwell RJ, Lodge M, Tozer GM, Baddeley H, Wilson I, Prise VE, Rustin GJS, Mount Vernon Hospital, Northwood, Middlesex, UK

CA4P dramatically reduces blood flow in animal tumours at non-toxic doses, causing haemorrhagic necrosis.

**Aim** To determine the dose and time response of CA4P on tumour blood flow in rats and humans.

**Methods** The absolute blood flow of tumours and normal tissues in BD9 rats bearing P22 carcinosarcomas was measured using radiolabelled iodoantipyrine (IAP). Serial dynamic MRI scans were performed on the same tumour model to enable comparison of the MRI and IAP techniques. Patients in the C RC Phase 1 trial of CA4P also had serial dynamic MRI scans using the same protocol as the *in-vivo* experiments. 30 dynamic images were obtained at 11.9s intervals prior to, during and after intravenous injection of Gadolinium-DTPA. The MRI sequence used was a 3 slice spoiled gradient echo, with echo time 9 ms, repetition time 80 ms, flip angle 70° and slice width 10 mm. Regions of interest (ROIs) were drawn around whole tumours and in skeletal muscle. The signal intensity/time curve for these ROIs was analysed using a pharmacokinetic model[1], and an assumed arterial input function. 2 parameters, $K^{trans}$ (transfer constant)[1] and AUC[2] (area under the curve) derived from this analysis were used as a measure of blood flow.

**Results** Tumour blood flow measured by IAP in rats was reduced by 88% at 10 mg/kg 6 hrs post CA4P, and by 20% at 24 hrs. At 100 mg/kg blood flow reduced by 99% at 6 hrs and 24 hrs. In the same tumour model the MRI parameters $K^{trans}$ and AUC were reduced by 71% and 65% respectively at 10 mg/kg after 6 hrs, and by 33% and 36% at 24 hrs. At 100 mg/kg $K^{trans}$ and AUC were reduced by 83% and 90%, maintained at 24 hrs. $r^2$=0.82 for correlation of $K^{trans}$ or AUC and IAP data.

12 of 22 patients treated with 5 to 114 mg/m² CA4P had serial MRI scans. No significant changes were observed below 52 mg/m². In 7 patients treated at 88 and 114 mg/m² mean $K^{trans}$ and AUC were reduced by 41%* and 36%* respectively at 6 hrs post CA4P, and by 19% (NS) and 24%* at 24 hrs. The maximal reduction seen was 84% ($K^{trans}$) in a patient at 88 mg/m². No significant changes in $K^{trans}$ or AUC were seen in muscle. 4 of these 7 patients had pain in the tumour site after each dose of CA4P The dose limiting toxicity was ataxia at 114 mg/m². (* =p<0.05)

**Conclusions** There was a high degree of correlation between absolute blood flow changes measured by IAP and the MRI parameters (. The time course of action of CA4P is similar in rat and human tumours. CA4P reduces $K^{trans}$ and AUC in human as well as animal tumours at doses below the DLT.

1 Tofts PS, Brix G, Buckley DL et al (1999) *J Magn Reson Imaging* **10:** 223–232
2 Evelhoch JL (1999) *J Magn Reson Imaging* **10:** 254–259

### 1.8 STUDIES ON THE MECHANISM OF ACTION OF THE VASCULAR TARGETING AGENT COMBRETASTATIN A-4 PHOSPHATE

C Kanthou* & GM Tozer, Tumour Microcirculation Group, Gray Laboratory Cancer Research Trust, PO Box 100, Mount Vernon Hospital, Northwood, Middlesex, HA6 2JR, UK

Combretastatin A-4 phosphate (CA-4-P) is in clinical trials as a promising antivascular agent that targets the microtubule cytoskeleton by binding at the colchicine locus of tubulin and altering its polymerization dynamics. Previous studies demonstrated that CA-4-P exhibits selective toxicity toward the tumour vasculature and causes its selective shutdown in animal models (*Dark et al, 1997, Cancer Res 57, 1829; Tozer et al, 1999, Cancer Res 59, 1626*).

We investigated CA-4-P's mechanism of action in cultured human umbilical vein endothelial cells (HUVECs). The state of tubulin polymerization was evaluated by extracting dimeric and polymeric forms of tubulin followed by western blot analysis. In control confluent HUVECs, approximately 70% of the tubulin existed in a polymerized form. CA-4-P (0.1 μM; 30 min) caused total tubulin depolymerization. Vascular smooth muscle cells and breast carcinoma MDA-231 cells were significantly less sensitive to CA-4-P as about 20% of their tubulin remained polymerized after treatment with CA-4-P (1–100 μM; 60 min). CA-4-P caused rapid (within min) HUVEC retraction and membrane blebbing, a manifestation of toxicity associated with apoptosis, previously shown to be induced by CA-4-P (*Iyer et al, Cancer Res, 1998, 58 4510*). Cytoskeletal alterations affecting the dynamics of actin are thought to be associated with membrane blebbing and changes in endothelial function such as vascular permeability. Stress stimuli that disrupt microtubules are known to activate actin reorganization via signals through the GTP-binding protein, Rho. We investigated the involvement of actin and Rho in CA-4-P mediated morphological changes. Confluent HUVECs treated with CA-4-P in the presence or absence of the Rho kinase inhibitor HA1077 were fixed, permeabilized and stained with Texas Red-conjugated phalloidin. In control cells actin microfilaments were predominantly found along the cell margin with some fine actin filaments traversing the cells. CA-4-P induced the formation of stress fibres across the cell body and cells retracted forming intercellular gaps. Additionally a significant proportion of cells exhibited a "blebbing" morphology in which F-actin accumulated in the bleb perimeters and also formed a dense spherical network in the cytoplasm. Pre-incubation with HA1077 abolished CA-4-P-mediated blebbing as well as stress fibre formation. Pre-treatment with the myosin light chain kinase inhibitor ML7 or the myosin ATPase inhibitor BDM led to a significant increase in the proportion of blebbing cells in response to CA-4-P suggesting that this phenotype resulted from a misassembly of actomyosin stress fibres. These data suggest that CA-4-P targets endothelial microtubules and regulates signals involved in the reorganization of the actin cytoskeleton via activation of Rho and other pathways.

### 1.9 TUMOUR ERADICATION BY COMBINED ANTIBODY-DIRECTED AND ANTIVASCULAR THERAPY

RB Pedley*[1], SK Sharma[1], G Boxer[1], AA Flynn[1], R Boden[1], R Watson[1], J Dearling[1], SA Hill[2], CJ Springer[3] and RHJ Begent[1], [1]Oncology Dept, RF & UCLMS, London NW32PF; [2]Gray Lab Research Trust, HA62JR; [3]ICR, SM25NG, UK

Common solid tumours contain well- and poorly-vascularised regions, each posing different problems for therapy. Using phosphor image analysis we have shown that antitumour antibodies localize therapy efficiently in well perfused outer areas of the tumour, but less so in poorly perfused areas containing radio- and chemo-resistant hypoxic cells. The addition of a complementary or synergistic therapy is therefore required, and antivascular agents offer an attractive approach. We have studied two antivascular agents, 5,6-dimethylxanthenone-4-acetic acid (DMXAA) and combretastatin A4-P (CA4-P), in combination with antibody-targeted therapies, using colorectal xenograft models. While possessing different modes of action, both drugs caused selective shut-down of tumour vessels leading to hemorrhagic necrosis of all but an outer rim of well-vascularized cells, but do not affect subsequent tumour growth. Radioimmunotherapy alone (7.4–18.5 MBq $^{131}$I-anti-CEA antibody) produced no cures, but when combined with either DMXAA (27.5 mg/kg) or CA4-P (200 mg/kg × 2) the tumours were eradicated in 85% and 90% of mice respectively. When DMXAA was combined with Antibody-directed Enzyme Prodrug Therapy (25 U CPG2, 1500 mg/kg CMDA prodrug), tumour growth inhibition was again significantly enhanced with no increase in toxicity. Both antivascular agents are in Phase I trials, and we are currently optimizing these approaches for combined therapy in the clinic.

Supported by the Cancer Research Campaign.

### 2.1 RATIONAL SELECTION OF ANTISENSE KIRSTEN *RAS* OLIGONUCLEOTIDE BY MAPPING ACCESSIBLE SITES WITH RIBONUCLEASE H

PJ Ross*[1,2], HJN Andreyev[1,2], M George[1], F di Stefano[1], D Cunningham[2], and PA Clarke[1], [1]CRC Centre for Cancer Therapeutics, Institute of Cancer Research, and [2]Gastrointestinal Unit, The Royal Marsden Hospital, Downs Road, Sutton, SM2 5PT, UK

Kirsten *ras* (Ki-*ras*) mutations are frequently observed in colorectal cancers and associated with an increased risk of recurrence and reduced odds of survival. Codon 12 is the most frequent site for Ki-*ras* mutations, however we found that antisense (AS) oligonucleotides (ODN) complimentary to such mutations were of limited efficacy. Therefore, an AS Ki-*ras* ODN was rationally selected. Ribonuclease H (RNase H) cleavage sites were mapped on 3′-labelled Ki-*ras* exons 1–3 mRNA incubated with a library of random sequence 17-mer ODN and *E. coli* RNase H. This assay identified 2 highly accessible sites: nucleotides 380–401 and 413–422. Nucleotides 380–401 correlated with a computer and ribonuclease predicted hairpin loop. Ki-*ras* mRNA was measured in the human colon adenocarcinoma SW480 cell line after transfection with a panel of ODN targeting accessible sites identified by the screening protocol. The most effective ODN, PR4, resulted in greater than 90% reduction in Ki-*ras* mRNA 24 hours after treatment of the cells. In contrast, a scrambled sequence control ODN had no effect on Ki-*ras* mRNA expression. Neither AS nor scrambled ODN altered the expression of GAPDH mRNA. Inhibition of Ki-ras protein expression was observed 72 hours after treatment with PR4. No effect on the expression of N-Ras protein was detected. In addition, following repeat treatments of human colon cancer cells with PR4 phosphorylation of mitogen activated protein kinase was reduced. Such a reduction was not observed after treatment of human colon cancer cells with scrambled oligonucleotide. Proliferation of human colon cancer cells treated with PR4 was reduced compared to a scrambled ODN but no specific cell cycle effects were detected. VEGF secretion by human colon cancer cells was reduced by 44% following treatment with PR4 compared to scrambled ODN (p=0.012).

A rationally designed AS Ki-*ras* ODN significantly reduced Ki-*ras* mRNA and protein expression. AS inhibition of Ki-*ras* in human colon cancer cells reduced proliferation and VEGF secretion. These findings justify further evaluation of the activity of ODN PR4.

### 2.2 INHIBITION OF THE RECEPTOR GENE EXPRESSION BY A NOVEL ANTI-GENE APPROACH

*PW Hewett[1], EL Daft[1], C Laughton[2] and JC Murray[1], CRC Academic Dept of Clinical Oncology, City Hospital, and [2]Cancer Research Labs; Dept. of Pharmaceutical Sciences, University of Nottingham, Nottingham, UK

We are developing anti-gene approaches to block tumour angiogenesis through the targeted formation of triplex DNA. The Tie receptor tyrosine kinases (Tie-1 and Tie-2) are largely restricted to endothelium and play a critical role in angiogenesis, vascular remodelling and maintenance of vessel integrity. Disruption of the Tie receptors has been shown to inhibit angiogenesis and reduce tumour growth as a consequence. Based upon our recent characterisation of the 5′ regulatory regions of the human *tie* genes, we have identified several suitable conserved sequences that could be targeted to disrupt transcription through DNA triplex formation. In order to test this paradigm we have focused on the human Tie-1 promoter which contains two ideal ~20 bp homopurine sequences. These sequences represent tandem *ets* transcription factor binding sites (EBS) which we have shown through the use of mutated promoter-reporter constructs are essential for promoter activity. Using plasmid binding and electrophoretic mobility shift assays we have demonstrated specific triplex forming activity of a range of anti-parallel homopurine TFOs ($K_d$<$10^{-7}$M) targeted to these sequences at 37°C and physiological pH in the presence of 10 mM $Mg^{2+}$. In order to determine the activity of candidate TFO on *tie*-1 promoter activity, TFO were incubated with *tie*-1 luciferase reporter constructs overnight at 37°C (pH 7.0) in the presence of 10 mM $Mg^{2+}$. Reporter constructs were then co-transfected with a CMV control promoter into endothelial cells and their activity determined by dual luciferase assays. Candidate TFO targeted to the two EBS were found to inhibit up to 60% of promoter activity in comparison with control oligos. As the formation and stability of these DNA triplexes decreases in the presence of physiological (~150 mM) $K^+$ ion concentrations we are examining the potential of partially phosphorothioate-linked TFO to overcome this problem in order to assess their activity on endogenous *tie*-1 expression. As Ets transcription factors are important in the regulation of a number of endothelial-restricted genes that contain similar multiple EBS motifs this strategy may have broad applicability to cancer and a number of conditions involving inappropriate angiogenesis or endothelial dysfunction.

*This work is supported by the Cancer Research Campaign*

### 2.3 ADENOVIRAL TRANSFER OF P53 AND P16$^{INK4A}$ RESULTS IN PANCREATIC TUMOUR REGRESSION

P Ghaneh*[1], W Greenhalf[1], M Humphreys[1], D Wilson[2], N Lemoine[3], JP Neoptolemos[1], [1]Dept of Surgery, Liverpool University, Daulby Street, UK, [2]Introgen Therapeutics, Texas, USA, [3]ICRF, Hammersmith Hospital, London, UK

**Background** Pancreatic cancer has a very poor prognosis as most patients present with advanced disease. Current regimens of chemoradiation are only moderately effective. Novel approaches to treatment are required. A high proportion of pancreatic cancers are associated with loss of function of key cell cycle control genes$^{p16INK4a}$ and p53. Previous work has shown p16$^{INK4a}$ and p53 gene therapy to inhibit pancreatic tumour cell growth *in vitro*. The aim of this study was to demonstrate regression of pancreatic tumours *in vivo* following transfer of wild type p16$^{INK4a}$ and p53 genes.

**Methods** Replication deficient recombinant adenoviruses which expressed wild-type p53 or p16$^{INK4a}$ under the control of a CMV promoter and SV40 polyadenylation signal were used for gene transfer to tumours. An identical vector expressing luciferase was used as the control. The human pancreatic cancer cell line MIAPACA-2 was used to generate subcutaneous tumours in 8-week old female nunu mice. Each mouse received an injection of $1 \times 10^7$ cells into each flank. Tumours were left to develop for four weeks. Each tumour then received one injection of $5 \times 10^{11}$ virus particles for three consecutive days. Tumours were removed from a single animal in each group 24 hours after the last injection for expression analysis. RT-PCR was used to amplify the transduced gene in tumours. Tumour volume was measured twice a week until the mice were sacrificed at 3 weeks. Treatment groups (consisting of 6 animals with 12 tumours) received intratumoural injections of virus expressing either luciferase, p53 alone or p16$^{INK4a}$ alone.

**Results** Wild-type p16$^{INK4a}$ and p53 RNA expression in the tumours was demonstrated using RT-PCR. There was a significant difference in tumour volume change between the treatment groups and the control group p<0.01 (Mann Whitney U), but not between groups with treated tumours.

**Conclusions** Transfer of wild-type p16$^{INK4a}$ and p53 genes produced significant regression of tumours in this animal model. These results indicate a role for tumour suppressor gene therapy in the treatment of pancreatic cancer.

### 2.4 NOVEL APPROACHES TO DESTROY MINIMAL RESIDUAL CANCER IN THE MUSCLE BED

RE Oakley*[1], E Phillips[1], D Wilson[2], and M Partridge[1], [1]Maxillofacial Unit/Oncology, Kings College Hospital, London SE5 8RX, UK, [2]Introgen Therapeutics Inc, 2250 Holcombe Boulevard, Houston, Texas 77030, USA

Gene transfer offers the possibility of novel therapies for head and neck squamous tumours. Since all adjuvants are most successful in the minimal residual disease setting, one potential application for these approaches is to eliminate nests of tumour which persist at the surgical margins after treatment. To date, most preclinical studies using adenovirus-mediated gene delivery have used subcutaneous rodent tumour models. However, residual tumour is frequently present in the muscle bed and it may be more difficult to transduce tumour cells at this site. The effect of 3 isogenic vectors, AD5CMVp53, Ad-βgal and Ad-empty, on tumour cell proliferation was determined by *in vitro* growth assay. A rodent model of minimal residual cancer in the muscle bed was established by implanting murine squamous cell carcinoma cells, harbouring a p53 gene mutation, into the muscle of 7–8-week-old syngeneic hosts. Subcutaneous tumours similar to those used in earlier preclinical studies were created over the dorsal flanks as comparitors. Nests of tumour were apparent 4 days after inoculation. Tumour nests in the muscle bed (minimum 6 mice per group) were injected with adenoviral particles on days 4, 7 and 9, with $5 \times 10^{10}$ vp Ad5CMVp53, Ad-βgal or Ad-empty, and histological examination performed on day 11. *In vitro* transduction of cells following infection with Ad-βgal approached 100% and $5 \times 10^{10}$ vp Ad5CMVp53 significantly reduced tumour-cell growth and induced apoptosis. Expression of the transgene was demonstrated by reverse transcription-polymerase chain reaction and Western blotting. Expression of βgal was detected by X-gal staining in approximately 20% of the cells within subcutaneous tumours, although the frequency of tumour cell transduction was much lower when treating nests of tumour in the muscle bed. Ad5CMVp53, delivered by direct intratumoural injection, had no effect on tumour cell progression in the rodent model of residual cancer in the muscle bed. These data show that it is feasible to develop rodent models of minimal residual cancer in syngeneic hosts. However, the present schedule utilising Ad5CMVp53 was not effective and the most likely explanation, based on the pattern of βgal expression, is that insufficient vector reaches the tumour nests in the muscle. These studies highlight the need to improve delivery of adenoviral-mediated gene transfer to muscle tissues, or to use combination therapies, without overlapping toxicities to increase selective and specific tumour cell killing.

### 2.5 FINE NEEDLE ASPIRATION CYTOLOGY OF BREAST CANCERS YIELDS SUFFICIENT MATERIAL TO MONITOR P53 BY WESTERN BLOTTING

HM-L Ball[1], TR Hupp[2], JP Blaydes[2], NM Kernohan[2], AM Thompson*[1], [1]Departments of Surgery & Molecular Oncology and Molecular & Cellular Pathology, University of Dundee DD1 9SY, UK

Fine needle aspiration (FNA) cytology is routinely used in the diagnosis of breast cancer as the least invasive method of in vivo breast cancer sampling. While FNA has been used for PCR studies, we sought to assess the feasibility of using FNA as a tool for monitoring functional p53 protein biochemistry (and p21 and p27, downstream effectors of p53) in vivo. P53 appears to be important in the pathogenesis of breast cancer and in the response of cancers to therapy; p53 status is now entering the clinical arena in clinical trials as the basis for treatment selection and as a therapeutic avenue.

For each of 14 histologically proven breast cancers, preoperative FNA (10 passes of a 19 g needle), specimen FNA (10 passes of a 19 g needle taken at immediate pathology cut-up) and frozen cancer tissue were lysed to extract protein for western blot analysis. Blots were probed with DO12 (which recognises a cryptic epitope in the core domain of p53) for absolute levels of p53 and for p53 phosphorylation sites using DO1 (recognising the N-terminus influenced by phosphorylation of serine 20) and FP3 (for serine 395 phosphorylation). Samples were also probed for p21 and p27 using in-house antibodies.

The protein extracted from the FNA samples was sufficient for at least five western blots. Using DO12, p53 protein was present in 11/14 cancers. Using DO1, 10/14 cancers were positive and with FP3 1/14. For target genes of p53, 9/14 cancers were p21 positive, but p27 detected in only 1/14. Analysis did not differ between the preoperative and postoperative FNA samples for any of the 14 cancers, suggesting intraoperative handling did not influence the p53 epitopes or downstream genes examined. However, p53 degradation products were observed in the frozen cancer samples.

FNA in vivo yields adequate protein for western blotting which could be used to monitor p53 activity during anticancer treatment. Furthermore, FNA studies of p53 may provide a more appropriate assessment of p53 function than using frozen tissue samples.

### 2.6 USE OF $O_2$-REGULATED BACTERIAL TRANSCRIPTION FACTORS TO TARGET GENE EXPRESSION TO HYPOXIC SITES IN TUMOURS

SG Sumner*[1], J Green[2], S Naylor[3] and CE Lewis[1], Departments of [1]Pathology, and [2]Molecular Biology and Biotechnology, Univ. of Sheffield, and [3]Oxford BioMedica, Oxford Science Park, Oxford OX4 4GA, UK

Recent advances in cancer gene therapy have involved the use of various molecular mechanisms to restrict gene expression to specific cells or sites within solid tumours. Specific DNA sequences, called hypoxia response elements (HREs), derived from oxygen-regulated mammalian genes, have been incorporated into therapeutic DNA constructs to ensure their expression only occurs in the low oxygen sites found in tumours. Hypoxic conditions promote the binding of a family of transcription factors, hypoxia-inducible factors (HIF) 1–3, to HREs, thereby promoting transcription. However, HREs exhibit low levels of activity in the mild hypoxia found in some normal tissue beds. We have therefore designed an ‘aerobic brake’ which could be engineered into HIF-activated constructs to ensure activation only in severe hypoxia. We propose to use a redox-responsive bacterial transcription factor (FLP; Fumarate-Nitrate reduction Regulator protein (FNR) Like Protein) from *Lactobacillus casei*, as such a brake. In this system, an FLP response element (FLP-RE) is introduced into a promoter. FLP operates a simple redox switch based on the interconversion of dithiol (inactive) and disulphide (active) forms, and is inactivated by virtual anoxia in *E. coli*. We hypothesise that oxidised FLP would repress expression of the reporter in normoxia or mild hypoxia by physically blocking the assembly and/or progress of the transcription complex, and that this repression would be removed under anoxic conditions, when FLP is converted to its reduced form. To assess the possible utility of FLP in gene therapy, expression plasmids were constructed, placing *flp* under the control of the IE-CMV promoter. Immunoblotting indicated that FLP was expressed in transiently transfected HT1080 cells, but requires the fusion of a Nuclear Localisation Signal (from EIA adenovirus protein) for efficient transport to the nucleus. The addition of several amino acids to the N-terminus of FLP did not compromise its activity in bacteria and the oxidised form of FLP is generated in mammalian cells. Furthermore, bandshift assays showed that bacterial FLP binds to an FLP-RE cloned into a mammalian promoter. Ongoing co-transfection studies with a reporter plasmid containing single or multiple FLP-Res just downstream of the TATA box will test the ability of FLP to repress gene expression in aerobic conditions. An alternative approach is to use FLP or related factors to control gene expression in such bacteria as *Salmonella*, which have been engineered to target and accumulate in solid tumours. We will show that the endogenous FNR induces reporter gene expression under anoxia in *Salmonella typhimurium*. Taken together, our data indicate that such bacteria-derived mechanisms could possibly be used to target high-level expression of therapeutic genes (delivered by viral, non-viral, cell-based, or bacterial vectors) to hypoxic/anoxic tumour regions.

### 2.7 THE SIGNIFICANCE OF MISMATCH REPAIR- AND P53 DEPENDENT APOPTOSIS IN THE SMALL INTESTINE,

OJ Sansom*[β], NJ Toft[β], DJ Winton[α], AR Clarke*, *=Dept of Biosciences, University of Cardiff, [α]=Dept of Oncology, University of Cambridge, [β]=Dept of Pathology, University of Edinburgh, UK

We and others have previously shown that Msh2 and p53 play a role in the initiation of apoptosis following DNA damage. However the significance of this to long term survival is still unclear. For example, after gamma irradiation, when there is clear p53 dependent apoptosis, p53 deficiency has not been reported to lead to increased clonogenic survival or frequency of mutation in p53 null mice. We have now studied these endpoints in relation to the mismatch repair gene Msh2. After treatment with the methylating agents temozolomide and MNNG, we observe Msh2 dependent apoptosis and, in the absence of Msh2, we see a significant increase in the mutation frequency as scored at the Dlb1 locus. In stark contrast the methylating agent MNU does not elicit MMR dependent apoptosis, but deficiency of Msh-2 does lead to increases in both clonogenic survival and mutation frequency. We have also analysed the Msh2 dependency of these responses with respect to cisplatin exposure, and find weak Msh-2 dependency in the apoptotic response but Msh2 dependency in either clonogenic survival or mutation frequency. This latter result is of particular interest as Msh2 has previously been reported to recognise the cisplatin adduct.

For all of the treatments listed above we find that the immediate apoptotic response is p53 dependent, but we only observe a p53-dependent increase in clonogenic survival following treatment with cisplatin.

In conclusion, we show that loss of Msh2 or p53 dependent apoptosis can lead to both an increase in clonogenic survival and an increase in mutation frequency, probably as a consequence of the loss of the apoptotic pathway. However, despite the attractiveness of the hypothesis that loss of apoptosis leads directly to increased survival and thus higher levels of mutation, for many circumstances this is clearly not the case.

### 2.8 NOVEL RESPONSE OF THE P53 PATHWAY TO TAMOXIFEN IN BREAST CANCER

D Ziyaie*[1], KL Ball[1], TR Hupp[2], A Ingram[1], AM Thompson[1], [1]Dept of Surgery & Molecular Oncology, [2]Dept of Molecular & Cellular Pathology, University of Dundee DD1 9SY, UK

The anti-oestrogen tamoxifen remains the first-line endocrine treatment of choice for women in whom tumours are positive for oestrogen receptors. However, only a proportion of these patients respond effectively to primary tamoxifen treatment. Some 50% of patients with oestrogen receptor positive tumours will respond to tamoxifen, but many who initially respond to primary tamoxifen treatment will eventually relapse and exhibit resistance to tamoxifen while on continuing treatment.

Although it is believed that anti-oestrogens such as tamoxifen exhibit both cytostatic and cytotoxic properties, inducing cell cycle arrest and apoptosis respectively, the exact mechanism by which tamoxifen inhibits tumour growth and molecular changes that lead to tamoxifen resistance are not fully understood.

It is generally accepted that genetic mutation is fundamental to malignant transformation and in particular alterations of the p53 tumour suppressor gene are of clinical significance. p53 abnormalities occur in nearly half of all human tumours including breast cancer where mutation or over-expression of the protein is observed in up to 52% of cancers.

To investigate the relation of the anti-tumour properties of tamoxifen with p53 and p21 (the p53-transactivated target gene mediating p53-dependent cell cycle arrest), we have treated MCF-7, an oestrogen receptor positive wild-type (active) p53 and tamoxifen sensitive breast cancer cell line, with tamoxifen. Using western blot and FACS analysis we have demonstrated that, in response to tamoxifen, these cells exhibit a marked induction of p21 protein in the absence of p53 protein induction, corresponding to an increase in the sub-G1 population of cells, accompanied by an increase in G1 cell cycle arrest. p53 protein in tamoxifen treated cells, in contrast with MCF7 cells treated with adriamycin, was not phosphorylated at Ser392, a site whose modification is a sensitive probe for p53 activation in the absence of changes in p53 protein levels (ref).

These data suggest that p21 protein induction in response to tamoxifen is independent of p53 protein activation. Current studies are centred on determining unambiguously whether p21/p53 protein mediates, or is just responding to, the effect of tamoxifen.

Jeremy P Blaydes and Ted R Hupp (1998) DNA damage triggers DRB-resistant phosphorylation of human p53 at the CK2 site. *Oncogene* **17:** 1045–1052

### 2.9 *p53* MUTATIONS – AN EARLY EVENT IN AIDS-ASSOCIATED NON HODGKIN'S LYMPHOMAS (NHL)?

A Reddy*[1], T Crook[2], I Weller[3] and DH Crawford[1], [1]Basic & Clinical Virology Group, University of Edinburgh, EH9 1QH, [2]LICR, London W2 1PG, [3]University College London Hospital, London WC1 6AU, UK

Chromosomal aberrations are a common feature of lymphomas associated with Human immunodeficiency virus (HIV) infection. AIDS-NHL develops in around 5–10% of HIV-infected individuals and involves deregulation of proto-oncogenes such as *c-myc, bcl-2, bcl-6 and ras*, as well as functional inactivation of tumour suppressor genes, *p53 and p16*. Although the contribution of these genes to lymphomagenesis has been well characterised, relatively little is known about prelymphomatous molecular events that precede clinical presentation in these individuals.

Persistent generalised lymphadenopathy (PGL) is an early manifestation of HIV infection and is characterised by hyperplasia and progressive fragmentation of the B-cell follicles, leading to end-stage lymphocyte depletion. We decided to analyse tissue from 24 HIV-infected individuals with PGL for early defining genetic abnormalities.

The *p53* gene is mutated in 50% of solid tumours and in 30–40% of AIDS-NHL. Therefore, we investigated whether alterations in the gene occurred early in these individuals. Mutations were detected in 3/24 (12.5%) PGL samples at codons 249 (G:T transversion), 238 (T:G transversion) and 229 (T:C transversion), by SSCP-PCR and sequencing. In contrast, the gene was intact in 16 control reactive lymph nodes and tonsils from HIV-uninfected individuals. This finding contradicts the popular observation that p53 mutation is a late event, and suggests that in the context of HIV infection structural lesions affecting the p53 gene are biologically important.

### 2.10 A NOVEL GERMLINE 7 BASE PAIR INSERTION IN P53; A FUNCTIONAL STUDY OF THE RESULTING TRUNCATED PROTEIN

Jodie Rutherford*[1], Carol E Chu[2], Ruth S Charlton[2], Paul Chumas[2], Graham R Taylor[2], Xin Lu[4], Diana Barnes[3], Richard Camplejohn[1], [1]Richard Dimbleby Dept., St Thomas' Hosp., SE1 7EH; [2]St James's University Hosp., Leeds, LS9 7TF; [3]ICRF Clin Oncol Unit, Guy's Hosp., SE1 9RT; [4]Ludwig Institute, St Mary's Hosp., W2 1PG, UK

The *p53* gene from a patient with a history of an osteogenic sarcoma and a choroid plexus papilloma was sequenced for the presence of a germline mutation. A novel heterozygous mutation was detected in exon five of p53 and was found to be a 7 base pair insertion after nucleotide 13160 resulting in a frameshift producing a stop codon at position 182. Various tests were carried out on this mutation in order to characterise it in functional terms. The apoptotic assay (Camplejohn et al. *Br J Cancer* 72: 654, 1995) which measures the amount of apoptosis in lymphocytes before and after irradiation gave a normal result (46% apoptosis, which is well within the normal range of 30–60%). The FASAY (Flaman et al. *PNAS* 92: 3963, 1995), which is a yeast based functional test for transactivation of targets by p53, also showed a wildtype result when using mRNA from the patient (100% white colonies). The 7 base pair mutation was synthesised by site-directed mutagenesis and was tested in the FASAY, this time a mutant result (100% red colonies) was obtained. Failure of the FASAY to detect the mutation using RNA from the patient's own cells appears to be due to the absence or very low level of mutant RNA in these cells. Transfection of this mutation into Saos-2 cells, showed the production of a truncated protein (24 kD on 12% SDS PAGE), which remained partially apoptotically active (70% wildtype). A similar report of retention of apoptotic function by a truncated protein has been published (Haupt et al. *Genes & Develop* 9: 2170, 1995). In the current study the mutated protein could not transactivate target genes nor suppress the growth of Saos-2 colonies. However, the truncated protein retained the ability to induce apoptosis in Saos-2 cells and the patient's apoptotic response was also normal. The possibility that apoptosis induction occurs by p53 induced transrepression of target genes was tested using an SV40-based target construct. However, the 7 base pair insertion mutant was non-functional in this assay also. Thus the mechanism by which this mutant protein induces apoptosis appears to be novel.

### 3.1 GASTRIC MALT LYMPHOMA: RESPONSE TO ANTI-HELICOBACTER THERAPY IN THE ONGOING LY03 RANDOMISED CO-OPERATIVE TRIAL OF OBSERVATION VS CHLORAMBUCIL AFTER ANTI-HELICOBACTER THERAPY

E Zucca, E Roggero, P Smith, C Traullé, C Copie-Bergmann, JC Delchier, R Souhami, F Cavalli and B W Hancock*, For the IELSG, GELA and UKLG

Eradication of Helicobacter pylori infection has been reported to induce histological regression in the majority of gastric MALT lymphomas. The International Extranodal Lymphoma Study Group (IELSG), the Groupe d'Étude des Lymphomes de l'Adulte (GELA) and the United Kingdom Lymphoma Group (UKLG) are conducting a cooperative trial (LY03) to ascertain for how long tumor regression is maintained, and whether the addition of single-agent chlorambucil is of benefit in those patients who respond to anti-helicobacter therapy.

From March 1995 to November 1999, 217 patients (39 from the UK) with localised, histologically reviewed low grade MALT lymphoma of the stomach were registered – 115 men and 102 women, with a median age of 68 years (range 20–85). 88% were positive for H. Pylori at diagnosis. Clinical presentations included pain/dyspepsia (79%), anorexia (16%), nausea/vomiting (15%) and haemorrhage (10%). The main sites of disease were antrum (55%), corpus (37%) and fundus (21%). So far 189 patients have been evaluated for response to anti-helicobacter therapy; histological regression of the gastric lymphoma was observed in 133 patients (70%, 95% C.I., 63% to 77%) with 105 (55%) complete and 28 (15%) partial remissions. The median time to lymphoma regression was 7 months. It appears that anti-helicobacter therapy can be safely given provided that a strict follow up is carried out. At a median follow up of 26 months there have been 15 relapses of gastric lymphoma (7%), most often with no evidence of H. Pylori reinfection. Histological transformation was observed only in 2 relapsed patients. Five patients have died so far, 1 of progressive lymphoma after high grade transformation, 2 of solid cancers (lung and melanoma) and 1 each of coronary thrombosis and pulmonary embolism. This study is ongoing; so far 80 patients have been randomised on the chlorambucil vs. observation trial.

### 3.2 OPTIMUM CONDITIONS FOR RADIOIMMUNOTHERAPY OF NON-HODGKIN'S LYMPHOMA (NHL) USING CAMPATH-1H MONOCLONAL ANTIBODY (MAB)

P Hadjiyiannakis*[1], G Hale[2], R Clutterbuck[1], S Eccles[1], P Carnochan[1], C Lebozer[1] VR McCready[1], D Catovsky[1], A Horwich[1], MJS Dyer[1], 1: The Institute of Cancer Research and 2: The Therapeutic Antibody Centre, Oxford, UK

**Background** The unconjugated humanised IgG1 CD52 Mab, CAMPATH-1H is effective against B- and T-cell chronic leukaemias. It has reduced efficacy against lymphomatous nodes, which may be treated with radioimmunotherapy. Tumour localisation of radiolabelled Mabs is improved by pre-treating patients with the unlabelled Mab. The reasons for this improvement remained unclear.

**Aim** To determine the criteria for optimum radioimmunotherapy of NHL.

**Methods** CAMPATH-1H was radiolabelled with $^{111}$Indium (In) using DOT Amaleimide as the chelating agent. The radiochemical purity was ≥95% with immunoreactivity ≥80%. The radiolabelled Mab was stable in plasma. CD52+ve human lymphoma xenograft models were raised subcutaneously in SCID/Nod mice. The biodistribution of 8 µg $^{111}$In-CAMPATH-1H, either alone, or in the presence of various other antibody constructs, was compared in these models.

**Results:** Mean tumour uptakes (expressed as % injected activity/gram) at the 48 hour time point in the Wien133 B-cell model were: i) no pretreatment, 3.1 (SD 0.4); ii) pretreatment with a non-Fc binding non-specific Mab 2.1(SD 0.4); iii) with unmodified CAMPATH-1H 9.3 (SD 1.9); iv) with normal human immunoglobulin (hu-IG) 12.8(SD 1.5). The best therapeutic ratio was obtained by blockade with hu-IG (p<0.05 to <0.001 according to comparison). In a separate experiment, pretreatment with hu-IG was equivalent to pretreatment with an Fc-binding non-specific Mab. Blockade with 500 µg of hu-IG improved the biodistribution as compared to blockade with 44 or 200 µg (p<0.05). Repeated doses of hu-IG (at –24 hours, 30 minutes, and + 48 hours) improved the therapeutic ratios as compared to a single dose at –30 mins. Only the radiolabelled specific antibody gave a favourable therapeutic ratio, with two radiolabelled non-specific antibodies failing to do so. The time course data showed that tumour uptake exceeded blood uptake at 36 hours and other normal tissue uptake at 16 hours. The maximal therapeutic ratios were achieved after 48 hours and persisted beyond that time point.

**Discussion** Blockade of Fc receptors markedly improved the tumour uptake and therapeutic ratio of the radiolabelled Mab. Blockade with native Mab was less good, presumably because of competition at the antigen binding site. Normal hu-IG is much cheaper and more readily available than Fc-binding non-specific Mabs. The time course for accumulation into lymphomatous masses suggest that for indium/yttrium a two step targeting approach (e.g. with streptavidin/biotin) should be used to optimise tumour delivery. Alternatively, a radioisotope with a longer half-life should be employed. These strategies will be explored in patients with NHL.

### 3.3 BEXXAR™ (IODINE-131 TOSITUMOMAB) RADIO IMMUNOTHERAPY FOR PATIENTS WITH B-CELL NON-HODGKIN'S LYMPHOMA

I Micallef, J Radford, K Britten, S Owen, D Deakin, H Jan, R Foley, R Barlow, B Carrington, J Lawrance, S Vinnicombe, M Harris, A Norton, A Lister, A Rohatiner, St Bartholomew's Hospital, London and Christie Hospital, Manchester, UK

Between July 1998 and November 1999, 40 patients (pts.), median age 53 years (range 27–81 years) received Iodine-131 tositumomab therapy (Bexxar™, Coulter Pharmacuetical Inc. and SmithKline Beecham) at two centres. 22 pts. were in a Phase II study, 18 on a compassionate release basis. Treatment comprised a single dosimetric dose, followed by three whole body gamma counts obtained over 7 days, to allow individualisation of the therapeutic dose. This was given 7–14 days after the dosimetric dose. 33 pts. had follicular lymphoma (FL), 4 pts. mantle cell lymphoma (MCL) and 3 pts. lymphoplasmacytoid lymphoma (LPC). In 7 pts., the disease had transformed to diffuse large B-cell histology. All had received prior treatment (median 2, range 1–18); 6 had received Rituximab (anti-CD20) and 4 high-dose therapy (HDT) supported by autologous haematopoietic progenitor cells. At the time of treatment, 18 pts. were in first progression, 10 in second, 10 in ≥ third and 2 had refractory disease. In 14 pts. the bone marrow was infiltrated at the time of treatment (<25%). Response is currently evaluated at 33 pts., 7 have not yet reached the point of first evaluation. The overall response rate (RR) at 7 weeks was 64% (21/33), complete remission (6), partial remission (15) pts. The RR according to histological subtype was: FL 74% (20/27), MCL 0 of 4, and LPC 1 of 2. Five of the 7 pts. with transformed disease are evaluable, 2 responsed and 3 progressed. Three of 4 evaluable pts. who had previously received Rituximab progressed. Three of 4 evaluable pts. who had previously received Rituximab received HDT are evaluable; 2/3 responded. The principal toxicity was haematological: platelets $\leq 20 \times 10^9$/1: 15%, neutrophils $\leq 0.5 \times 10^9$/1: 20%, Hb ≤10 g/dl: 30%. The nadir typically occurred at week 5–6; blood counts recovered by week 8–9. No patients developed HAMA following treatment. At a median follow-up of 6 months (1.5–15 months), 5/21 pts. have progressed. These results demonstrate that Bexxar™ radioimmunotherapy is a safe and effective treatment for patients with recurrent B-cell non-Hodgkin's lymphoma.

This study was supported by the ICRF, CRC and both NHS Trust.

### 3.4 M-FISH ANALYSIS AND DETECTION OF CHROMOSOME ABNORMALITIES PRE-TRANSPLANTATION IN PATIENTS DEVELOPING THERAPY-RELATED MDS AND SECONDARY AML FOLLOWING HIGH DOSE TREATMENT FOR NON HODGKINS LYMPHOMA

DM Lillington*, INM Micallef, E Carpenter, MJ Neat, JAL Amess, J Matthews, NJ Foot, AZS Rohatiner, BD Young, TA Lister, ICRF Department of Medical Oncology and Department of Haematology, St. Bartholomew's Hospital, London ECIM 6BQ, UK

Therapy-related myelodysplasia (tMDS) and secondary acute myeloid leukemia (sAML) represent significant late complications in the treatment of primary malignancies. In patients with non-Hodgkins lymphoma (NHL), the incidence of tMDS/sAML following high-dose therapy (HDT) is between 5 and 15%. Twenty-eight of 230 patients with NHL who received cyclophosphamide and total body irradiation supported by autologous haematopoietic progenitor cells at St. Bartholomew's Hospital have developed tMDS/sAML. The majority showed complex karyotypes with complete or partial loss of chromosomes 5 and/or 7. Multi-colour fluorescence *in-situ* hybridisation (M-FISH) was used to characterise complex rearrangements and revealed cryptic changes not identified on routine G-banded analysis. To address whether pre-transplant or transplant related factors play the critical role in the development of tMDS/sAML, FISH analysis was undertaken to look for abnormalities consistent with tMDS/sAML in cryopreserved bone marrow samples taken before transplant. Significant levels of abnormal cells were found *before HDT* in 19 of 20 patients screened and who subsequently developed tMDS/sAML. Prior cytotoxic therapy, therefore, plays an important aetiologic role and may predispose to the development of tMDS/sAML. Using a triple FISH assay these 'at risk' patients can be identified before proceeding to HDT. This could have major implications for the management of such patients.

### 3.5 REPAIR OF DNA INTERSTRAND CROSSLINKS AS A MECHANISM OF CLINICAL RESISTANCE TO MELPHALAN IN MULTIPLE MYELOMA

VJ Spanswick*[1], C Craddock[2], M Sekhar[3] and JA Hartley[1], [1]CRC Drug-DNA Interactions Research Group, Dept of Oncology, University College London and Royal Free Medical School, 91 Riding House Street, London WIP 8BT, [2]The Cancer Centre, Queen Elizabeth Hospital, Edgbaston, Birmingham B15 2TH, [3]Dept of Clinical Haematology, West Middlesex University Hospital NSH Trust, Isleworth TW7 6AE, UK

High dose melphalan therapy followed by autologous peripheral blood stem cell (PBSC) transplantation is considered to be an effective treatment regime for relapsed multiple myeloma. However, despite its success nearly all patients relapse and become refractory to further melphalan treatment. The basis of this clinical drug resistance is unclear. Mechanisms of resistance to melphalan studied *in vitro* include alterations in drug transport, detoxification and inhibition of drug-induced apoptosis. An additional important mechanism is enhanced repair of drug-induced damage which may result in resistance to nitrogen mustard based therapies in some haematological malignancies such as chronic lymphocytic leukaemia.

The single cell gel electrophoresis (Comet) assay was used to assess the formation and repair of melphalan-induced DNA interstrand crosslinks (ICL), the critical lesion associated with melphalan cytotoxicity. Isolated plasma cells from bone marrow of multiple myeloma patients were treated *ex vivo* with melphalan (0–100 µM) for 1 hr followed by 16 hr drug-free post incubation to allow maximum formation of ICL. Repair of ICL was assessed a further 24 hr later. Initially two patient populations were studied; those who were melphalan naïve and those known to be clinically melphalan resistant. ICL formation was found to be similar in both patient populations suggesting that cellular uptake and detoxification mechanisms may not have a role in the development of clinical resistance. Patients previously untreated with melphalan demonstrated no evidence of ICL repair. In contrast, in a resistant patient who had previously failed an autologous PBSC transplant following melphalan therapy, all ICL were repaired within 24 hr. This evidence suggests that enhanced repair of melphalan-induced ICL may be the major mechanism for development of clinical resistance. Using the Comet assay it may be possible to detect the emergence of a resistance phenotype, measure heterogeneity of response within the patient populations, and ultimately predict the outcome of melphalan therapy in multiple myeloma.

### 3.6 DEMONSTRATION OF FUNCTIONAL DNA MISMATCH REPAIR IN LYMPHOBLASTS FROM PATIENTS WITH LEUKAEMIA

EC Matheson*[1], AG Hall[1], G Marra[2], [1]Molecular Pharmacology Laboratory, Cancer Research Unit, Medical School, Framlington Place, Newcastle upon Tyne, NE2 4HH, UK, [2]Institute for Medical Radiobiology, August Forel-Strasse 7, 8008 Zurich, Switzerland

DNA mismatch repair (MMR) plays an important role in maintaining the integrity of the genome by repairing base-base mispairs and insertion/deletion loops that may arise during normal DNA replication. Defects in the MMR system result in a mutator phenotype and a susceptibility to cancer. In addition, MMR defects have recently been identified as a cause of drug resistance. As current treatment protocols for leukaemia commonly involve the use of etoposide, doxorubicin and thiopurine drugs, all of which have been shown to be tolerated by cells which are defective in MMR, we have analysed the protein expression of the main components of this pathway in a range of leukaemias by immunoblotting and determined the functionality of the protein using a mismatch repair assay.

A total of 12 lymphoblast samples from patients with acute lymphoblastic leukaemia (ALL), acute myeloid leukaemia (AML) and chronic myeloid leukaemia (CML) were analysed for expression of key mismatch repair proteins, hMLH1, hPMS2, hMSH2, hMSH6 and PCNA by western blotting. All samples were comparable in terms of blast % (all >90%) and proliferative activity. Cytoplasmic extracts were also freshly prepared from each of the samples and the efficiency of these extracts to repair DNA mismatches was tested using M 13mp2 heteroduplex substrate containing a G.T mispair using an assay for *in vitro* MMR (Thomas, D.C., Umar, A., Kunkel, T.A. 1995. A Companion to Methods in Enzymology, Vol. 7, 187–197).

hMLH1, hPMS2 and hMSH2 proteins were highly expressed in all of the leukaemic samples studied, however there was a wide variation in repair efficiency, ranging from less than 1% in the case of a relapse ALL, to greater than 90% in the case of a presentation ALL. There was no trend or correlation found with disease state, although as this is a prospective study, the analyses will be ongoing. Complementation studies using recombinant MutSα (hMSH2/hMSH6) and MutLα (hMLH1/hPMS2) protein and cytoplasmic extract from known mismatch repair deficient cell lines are currently being carried out on the samples.

To the best of our knowledge, this is the first time a mismatch repair assay has been carried out on primary human tumour samples. The results suggest that assessment by western blotting alone may be insufficient to determine mismatch repair status, at least in human leukaemias.

### 3.7 TIMING AND DOSE CHEMOTHERAPY ARE CRITICAL TO SUCCESSFUL OUTCOME WITH RADIOTHERAPY IN B-CELL LYMPHOMAS

J Honeychurch, AM Vandersteen, PWM Johnson, MJ Glennie, TM Illidge, CRC Department of Oncology, Cancer Sciences Division, Southampton General Hospital, Southampton University, SO16 6YD, UK

Radioimmunotherapy (RIT) has emerged as a highly promising new treatment for B-cell lymphomas and is now expected to play an important part in the management of these disease. However, a number of ongoing uncertainties remain, which include the most appropriate way to integrate this new treatment into current protocols. We have developed two in vivo models of B cell lymphomas and have specifically used them to investigate the importance of the timing of administering the alkylating agent Cyclophosphamide (CYC) before and after RIT.

Using A31 (CBA mice) and $BCL_1$ (BALB/c) models, animals were injected with $10^6$ cells and either given CYC on day 7 followed by RIT on day 14 or RIT on day 7 followed by CYC on day 14 and compared with the effects of RIT or CYC alone. The RIT given included a panel of $^{131}$I labelled antibodies (anti-Id [Immunoglobulin idiotype]; anti-MHC Class II, anti-CD40) previously reported as showing high therapeutic efficacy in the $BCL_1$ model (Illidge et al BLOOD 94:233–243). When given prior to RIT, higher doses of CYC (5 mg) ameliorated the therapeutic effects of RIT with anti-Id and anti-CD40 mAbs and survival was found to be similar to CYC alone. In contrast animals treated with lower doses (1–3 mg) of CYC after RIT in the $BCL_1$ model were long-term (>200 days) disease free survivors. The kinetics of CYC induced peripheral T and B cells depletion was investigated in vivo in both BALB/c and CBA mice and found to be dose dependent and to reach a nadir around day 7 when the RIT was administered. We believe that these depletion studies may explain the decreased efficacy of CYC prior to RIT and underlie the critical role that immune effector cells play in effective RIT. We conclude that the timing and use of immunosuppressive chemotherapy prior to RIT in B cell lymphomas may seriously affect the successful outcome of durable disease free survival and that these findings have important implications for implementing treatment strategies with RIT in the clinic.

### 3.8 DEXRAZOXANE PREVENTS THE EMERGENCE OF MULTIDRUG RESISTANCE IN THE HUMAN LEUKAEMIA LINE, K562

J Sargent*[1], C Williamson[1], C Taylor[1] and K Hellmann[2], [1]Haematology Research, Pembury Hospital, Kent TN2 4QJ, [2]Windleshaw House, Withyham, Sussex TN7 4DB, UK

Dexrazoxane combined with doxorubicin (+ 5-fluorouracil+cyclophosphamide – the FAC regime) leads to a significant decrease in doxorubicin cardiotoxicity and a significant increase in median survival time for patients with advanced breast cancer responsive to FAC. The reason for this increase in survival may be related to a reduction in the emergence of multidrug resistance (MDR). In order to test this hypothesis, we induced resistance to doxorubicin in the K562 cell line by growing cells in increasing concentrations of doxorubicin (10–40 nM) in the presence (20 and 200 nM) and absence of dexrazoxane. The doxorubicin sensitivity of all resultant sublines was measured using the MTT assay. Flow cytometry was used to assess the MDR phenotype, measuring P-glycoprotein expression with MRK 16 plus indirect immunofluorescence and drug accumulation in the presence and absence of PSC 833 for functional P-glycoprotein. Long-term growth in doxorubicin increased the cellular resistance ($IC_{50}$) of K562 cells in a concentration dependent manner ($r^2$=0.999). This increase in $IC_{50}$ was markedly impaired in the presence of dexrazoxane (p<0.001). In parallel, both the induction of P-glycoprotein expression and its function were significantly reduced in the presence of dexrazoxane (p<0.0001).

| | **$K562dox_{30}$** | **$K562dox_{30}$ +DXRz (20 nM)** |
|---|---|---|
| DOX $IC_{50}$ (μM) | 8.6 | 0.58 |
| Pgp (ratio: fluorescence test/control) | 16.5 | 2.05 |
| Drug accumulation (ratio: fluorescence +PSC/–PSC) | 17.2 | 1.07 |

These results suggest that concomitant treatment of patients with initially drug-sensitive disease with an agent such as dexrazoxane, might suppress the appearance of resistant MDR subclones so allowing the responders to continue to respond.

### 3.9 A POPULATION-BASED STUDY OF INCIDENCE AND MORTALITY FOR OSTEOSARCOMA IN ENGLAND AND WALES

PJ Jankowska*[1], P Babb[2], MJ Quinn[2], JS Whelan[1], [1]Meyerstein Institute of Oncology, Middlesex Hospital, London WC1E 6BA, [2]Office for National Statistics, 1 Drummond Gate, London SW1V 2QQ, UK

**Background** Accurate data about the epidemiology of rare cancers are not readily available. This study describes for the first time the incidence of and survival from osteosarcoma for England and Wales.

**Methods** Incidence data on 2931 histologically-confirmed osteosarcomas, as collated by the Office for National Statistics (ONS) between 1971 and 1993, were analysed by age, sex and site. Survival analyses (sub-classified by sex, deprivation category, time cohort and NHS region) were performed on the data available for children aged 0–15 and diagnosed before 1991.

**Results** The incidence of osteosarcoma in England and Wales was 4.1 per million in 1993, compared with 5.7 in 1971. The rates were slightly higher for males than females. A bimodal age distribution was confirmed 57% of cases arose in the lower limb and 13% in the upper limb. The percentage distribution in the pelvis/sacrum/coccyx, craniofacial bones, vertebral column (excluding sacrum and coccyx) and ribs/sternum/clavicle is 11%, 5%, 3% and 3% respectively. 6% of osteosarcomas had an unspecified site code. For children diagnosed in 1971–75 one-year survival was 55% and five-year survival 17%. Those diagnosed between 1986–90 had one-year survival of 88% and five-year survival 51%. The average increases in relative survival between successive five-year periods from 1971–75 to 1986–90 were 11% for one-year survival and 12% for five-year survival. There were no survival differences between boys and girls. Survival increased substantially in every NHS region. There were no statistically significant relations between survival and socio-economic deprivation.

**Conclusions** The fall in the incidence of osteosarcoma is most likely due to improved diagnosis, principally in older patients. The incidence data confirm previous reports of regional data from Europe and the SEER data in the USA. Improved one-year and five-year survival from osteosarcoma in children reflect more effective treatment. Further analyses of these data will be carried out to determine if these improvements of survival in osteosarcoma are maintained. Population-based data can provide valuable information about rare cancers. Such data may be useful in guiding future allocation of resources for health care provision and research but may be limited by data quality.

### 3.10 LONG-TERM SURVIVAL IN LOCALISED EXTREMITY OSTEOSARCOMA: MATURE FOLLOW-UP FROM TWO RANDOMISED TRIALS OF THE EUROPEAN OSTEOSARCOMA INTERGROUP

JS Whelan*[1], S Weeden[2], B Uscinska[2] and A McTiernan[1], On behalf of the European Osteosarcoma Intergroup, [1]London Bone and Soft Tissue Tumour Service, Middlesex Hospital, UCLH Hospitals NHS Trust, London WIN 8AA, [2]Cancer Division, MRC Clinical Trials Unit, 222 Euston Road, London W1N 2DA, UK

The European Osteosarcoma Intergroup (EOI) has completed two large randomised trials in localised extremity osteosarcoma. EOI BO02/80831 was open to accrual between 1983 and 1986, 179 eligible patients were randomised between two-drug (cisplatin and doxorubicin) and three-drug (cisplatin, doxorubicin and methotrexate) therapy. Between 1986 and 1993, 391 eligible patients were randomised into EOI BO03/80861 which compared the same two-drug regimen with prolonged multidrug therapy based on the T10 regimen.

Mature follow-up data is now available for these 570 patients. The median follow-up time is 9.6 years, 94% of patients have been followed to death or 5 years. 288 (51%) patients received two-drug therapy, 90 (16%) received three-drug and 192 (34%) multi-drug. The median age of patients was 16 (range 3–40) and 363 (64%) were male. Primary tumour site was femur in 315 (55%), tibia or fibula in 177 (31%) and upper limb in 78 (14%).

Limb salvage surgery was possible for 373 (65%) patients. Good histological response based on > 90% necrosis occurred in 30% of patients. Overall 5-year survival was 56% (95% CI 52–60%) and 5-year progression-free survival was 44% (95% CI 40–48%). There were no significant differences in survival based on trial, treatment, age or sex. Good histological response, tumours of the tibia or fibula and limb-conserving surgery were all associated with improved survival.

Site of first recurrence was local in 21 (4%), bone in 20 (4%), lung in 189 (33%) and combined for 58 (10%) patients. First recurrences after 5 years are uncommon – only 3 occurred in this patient group. Two patients died from chemotherapy-related toxicity more than 5 years after randomisation.

These data, collected prospectively for a large group of patients, provide important information on long-term survival for primary extremity osteosarcoma.

### 4.1 PREOPERATIVE CHEMORADIOTHERAPY IN CANCER OF THE OESOPHAGUS: EVIDENCE OF RADIATION AND CHEMOTHERAPY DOSE RESPONSE

JI Geh*[1], SJ Bond[2], SM Bentzen[3], R Glynne-Jones[2], [1]Queen Elizabeth Hospital, Birmingham; [2]Mount Vernon Hospital and [3]Gray Laboratory, Northwood, UK

**Introduction** Patients achieving pathological complete response (pCR) following preoperative chemoradiotherapy (CRT) for oesophageal cancer have improved survival. We identified 52 trials (46 phase II: 6 randomised) of preoperative CRT totalling 3078 patients; pCR was observed in 23%. Different radiotherapy doses & fractionations and chemotherapy drugs, doses & scheduling were prescribed in each trial which may account for the large differences in pCR rates achieved. The aim of this meta-analysis was see if a dose response relationship exists between radiotherapy & chemotherapy dose and pCR.

**Methods** Trials were included within this meta-analysis if they used a single radiotherapy dose/fractionation, used a single 5FU, cisplatin or mitomycin C based chemotherapy regimen and provided information on patient numbers, age, resection & pCR rates. Twenty six trials were eligible (1335 patients) of which 311 patients (24%) achieved pCR. The endpoint used was pCR and the following covariates were analysed; radiotherapy dose, radiotherapy dose × dose/fraction, radiotherapy treatment time, chemotherapy dose, median age of patients within trial. Multivariate logistic regression was used to predict the probability of pCR following preoperative CRT.

**Results** Increasing radiotherapy dose was associated with increasing pCR rates *(p=0.006)*. The alpha/beta ratio of oesophageal cancer was estimated to be 4.91 Gy (95% CI 1.48–16.68 Gy). Increasing radiotherapy treatment time reduced the probability of pCR *(p=0.035)*; estimated radiotherapy dose lost per day was 0.83 Gy (95% CI 0.25–1.39 Gy). There was also evidence of a chemotherapy dose response relationship for both 5FU *(p=0.003)* and cisplatin *(p=0.018)*; 1 gram/$m^2$ of 5FU estimated to be equivalent to 2.60 Gy (95% CI 1.10–7.21 Gy) and 100 mg/$m^2$ of cisplatin estimated to be equivalent to 11.1 Gy (95% CI 3.00–39.0 Gy). Mitomycin C dose did not seem to influence pCR rates *(p=0.60)*. The lower the median age of patients within the trial, the higher the tendency to achieve pCR *(p=0.019)*.

**Conclusions** There was significant evidence of a dose response relationship between increasing radiotherapy, 5FU and cisplatin dose and pCR. Additional significant factors were radiotherapy treatment time and median age of patients within the trial.

### 4.2 A PHASE II STUDY OF GEMCITABINE AND FLUTAMIDE IN ADVANCED PANCREATIC CANCER

A Mayer*[1], J Shaw[1], SD ‘Ath[1], P Price[2], C Blesing[3], P Corrie[1], [1]Oncology Centre, Addenbrooke’s NHS Trust, Cambridge, [2]Dept of Oncology, Hammersmith Hospital, London, [3]Dept of Oncology, The Churchill, Oxford Radcliffe Hospital, Oxford, UK

Patients with pancreatic cancer frequently present with advanced disease and life expectancy is measured between 3 and 9 months. Survival benefit of chemotherapy has so far been limited. Both, Gemcitabine (Burris *et al.*, 1997), a deoxycitidine analogue and Flutamide, an antiandrogen (Greenway, 1998) have been reported to improve median survival. We report the first study combining Flutamide and Gemcitabine in pancreatic cancer.

Patients with histologically proven non-resectable pancreatic cancer and measureable disease received Gemcitabine (1 g/$m^2$) on day 1, 8 and 15 of a 28 day cycle, while Flutamide 250 mg tds was given concurrently over the whole period. Tumour imaging was performed prior to entry into the study and subsequently after every two cycles.

So far, 15 patients (11 male, 4 female, median age 58, range 36–72 years) have been entered into the study. Performance status was 0, 1, and 2 in 6, 5, and 4 patients and tumour stage was II, III and IV in 3, 3, and 8 patients, respectively. The remaining patient was suffering from local recurrence after pancreatectomy. A median of 3 cycles of Gemcitabine was given (range 1–10). The best response was a partial response (PR) after 2 cycles in 2 and after 4 cycles in another 2 patients (response rate 27%). A minor response (MR) occurred in 1 patient after 5 cycles. Four patients showed stable disease (SD), 4 patients progressive disease (PD), 2 patients are currently awaiting their first reassessment CT scan. Median survival has not been reached yet. The full dose of Gemcitabine was given in 9 patients, dose modifications were necessary due to grade 3 neutropenia (1 patient), grade 3 anemia (1 patient), grade 1 thrombopenia (3 patients), and grade 3 hypersensitivity (1 patient). Flutamide was stopped due to grade 3 liver toxicity in 1 patient and due to grade 2 nausea/vomiting in a second patient. It was reduced to 250 mg bd in 2 patients due to grade 2 hot flushes in 1 patient and grade 2 nausea/vomiting in another patient. Further grade 3 toxicity included nausea/vomiting in 2 patients, while the majority of patients experienced only mild toxicity (grade 1/2 fatigue in 5 patients, grade 1/2 nausea/vomiting in 3 patients).

The response rate of 27% indicates that Gemcitabine in combination with Flutamide is a promising regimen in advanced pancreatic cancer with acceptable toxicity.

Greenway (1998), *Br Med J* **316:** 1935
Burris (1997), *J Clin Onc* **15:** 2403

### 4.3 A PHASE I TRIAL OF ANTIBODY DIRECTED ENZYME PRODRUG THERAPY (ADEPT) IN PATIENTS WITH ADVANCED COLORECTAL CARCINOMA OR OTHER CEA PRODUCING TUMOURS

RJ Francis*[1], SK Sharma[1], L Sena, C Springer[2], AJ Green[1], LD Hope-Stone[1], J Martin[2], KL Adamson[1], D O’Malley[1], E Tsiompanou[1], H Shahbakhti[1], S Webley[1], D Hochhauser[1], AJ Hilson[1], RHJ Begent[1], [1]Department Oncology, Royal Free and UCL Medical School, London NW3 2PF, [2]CRC Centre for Cancer Therapeutics, Inst. of Cancer Research, Surrey SM2 5NG, UK

Colorectal cancer is the second leading cause of cancer death in the UK. Conventional treatments remain unable to cure patients with advanced or metastatic disease, so there remains an urgent need to develop novel therapies.

In antibody-directed enzyme prodrug therapy (ADEPT) a prodrug is activated selectively at the tumour site by an enzyme targeted to the tumour by an antibody (antibody – enzyme conjugate). Previous clinical trials have shown evidence of tumour response[1,2]. However, effectiveness was limited by the long half-life of the activated drug, which diffused back into the circulation and caused myelosuppression. Also, the targeting system, although giving high tumour to blood ratios of antibody-enzyme conjugate (10,000:1) was excessively complicated, requiring administration of a clearing antibody in addition to the antibody-enzyme conjugate. The purpose of this study was to study tumour targeting of the antibody-enzyme conjugate without the clearing antibody, and to investigate a new highly potent prodrug (bis-iodo phenol mustard, ZD2767P) whose activated form has a short half-life.

Between Nov 1997 – Nov 1999 28 patients were recruited and 27 treated in the phase I ADEPT trial. The MTD of ZD2767P was reached at 15.5 mg/$m^2$ × 3 with a serum carboxypeptidase G2 (CPG2) level of 0.05 U/mL. Thrombocytopenia and neutropenia limited dose escalation. Other toxicities were mild. Without a clearing antibody this system achieved tumour: normal tissue ratios of antibody-enzyme conjugate of less than 10:1 (tumour biopsy and gamma camera) at the time of prodrug administration. The ZD2767P prodrug was shown to clear rapidly from the circulation, activated drug was not measurable in the blood, and evidence of drug effect was demonstrated by the presence DNA interstrand crosslinks in a tumour biopsy (comet assay).

Conclusion: The A5B7–CPG2 chemical conjugate requires a clearing antibody to achieve high tumour to normal tissue ratios that are required for therapy. However a genetic fusion protein of antibody – enzyme may solve this problem. ZD2767P prodrug was activated *in vivo* by CPG2 and has potential for use in ADEPT systems in which higher tumour to normal tissue enzyme ratios are achieved.

This work was done on behalf of the CRC Phase I/II Committee.

1 Bagshawe K et al (1995) *Tumour Targeting* **1:** 17–30
2 Napier M et al (2000) *Clin Cancer Res* (in press)

### 4.4 PHASE II STUDY OF IRINOTECAN AND HIGH DOSE INFUSIONAL 5-FU AND FOLINIC ACID (MODIFIED DE GRAMONT) FOR FIRST-LINE TREATMENT OF ADVANCED OR METASTATIC COLORECTAL CANCER

P Leonard,*[1] JA Ledermann,[1] R James,[2] D Hochhauser,[1] M Seymour[3], [1]Royal Free and University College Medical School, London, [2]Christie Hospital Manchester, [3]Cookridge Hospital Leeds, UK

**Aims** To investigate the response and toxicity of the combination of irinotecan with a modified de Gramont schedule of infusional 5-fluorouracil (5-FU) and folinic acid as first line therapy in advanced or metastatic colorectal cancer.

**Methods** Forty-two patients (16 F, 26 M) with a median age of 57 y (range 34–79 y) and WHO performance status 0–1 were treated. None had previously received chemotherapy except in the adjuvant setting. Every two weeks, patients received the following regimen: (a) irinotecan 180 mg/m$^2$ over 30 mins by iv infusion, (b) L-folinic acid 175 mg (or d, L folinic acid 350 mg) over 2 hrs by iv infusion (c) bolus iv injection of 5-FU 400 mg/m$^2$ followed by 5-FU 2400 mg/m$^2$ over 46 hrs by iv infusion. Three patients had 5-FU at the infusional dose of 2800 mg/m$^2$, but following two toxic deaths the dose was reduced to 2400 mg/m$^2$.

**Results** The median number of treatment cycles administered was 8 (range 1–12) with 31 patients completing at least 6 cycles and 16 patients completing 12 cycles. Dose reductions of both drugs were required in 10/42 (24%) patients because of side effects. In total 38/42 (90%) were evaluable for response. Of these, 11/38 (29%) patients had a partial response and a further 13/38 (34%) achieved disease stabilisation. Median survival times have not yet been established as the vast majority of patients are still alive. Haematological toxicity grade (Gd) 3/4 occurred in 7 (16%) patients, including two patients who died at the start of the study. Thrombocytopenia was not seen. Non-haematological toxicities included: diarrhoea Gd1/2 (28%), Gd3/4 (5%); nausea and vomiting Gd1/2 (23%); mucositis Gd1/2 (21%); alopecia Gd1/2 (33%); no patients reported Gd3/4 alopecia; dizziness and disorientation was experienced in two patients. Three patients were given atropine for cholinergic side effects.

**Conclusion** These data show that the modified de Gramont schedule in combination with irinotecan offers an active and well tolerated first line treatment for advanced or metastatic colorectal cancer. The overall toxicity was predictable, reversible and manageable, the main dose limiting toxicity was neutropenia. This regimen will now be used in a randomised study conducted by the Medical Research Council.

### 4.5 INDIVIDUALIZING TREATMENT APPROACHES WITH ANTIFOLATE ANTIMETABOLITES

[1]AL Jackman, [1]HER Ford, [1]D Cunningham, [1]DC Farrugia, [1]F Mitchell, [2]K Danenberg and [2]P Danenberg, [1]CRC Centre for Cancer Therapeutics, The Institute of Cancer Research, and the Royal Marsden Hospital Trust, Sutton, Surrey; [2]University of California, Los Angeles, USA

The specific antifolate thymidylate synthase (TS) inhibitor raltitrexed (RTX; Tomudex™) gives similar response rates to 5-fluorouracil (5-FU) and leucovorin or continuous infusion 5-FU in colorectal cancer. Preclinical studies suggest that RTX and 5-FU should have incompletely overlapping spectra of antitumour activity. This is also predicted from their different mechanisms of cellular uptake, metabolism and specificity for the target, TS. Although the toxicity profile of RTX is generally more acceptable than 5-FU/LV, a small percentage of patients treated with RTX develop a life-threatening combination of diarrhoea and neutropenia. Elevated levels of TS, thymidine phosphorylase (TP) and dihydropyrimidine dehydrogenase (DPD) are associated with non-response to 5-FU. Taken together, this information suggests a role for individualised therapy in colorectal cancer based on gene expression patterns or pharmacodynamic end-points. 29 patients were treated with RTX 3 mg/m$^2$ 3-weekly, and 24 underwent pretreatment (day –1) and post-treatment (day 5) biopsies of metastatic tumour (liver) and normal gastrointestinal mucosa. Pre-treatment biopsies were analysed for TS, folylpolyglutamate synthetase (FPGS), TP and DPD mRNA by RT-PCR. Pre- and post-treatment biopsies were stained for TS, Ki-67 (a proliferation marker) and dUTPase by immunohistochemistry. Plasma and tissues samples were assayed for RTX levels by radioimmunoassay, and plasma for 2′deoxyuridine (dUrd; a surrogate marker of TS inhibition) by HPLC. Most patients are now evaluable for response. Results have shown that low TS/β-actin mRNA ($\leq 4.1 \times 10^3$; previously defined cut-off for response to 5-FU) was significantly associated with response (5/6 responders compared to 1/11 non-responders; p=0.005). By contrast, high gut TS expression was associated with anti-proliferative toxicity. 6/9 patients with gut TS/β-actin $\geq 5.9 \times 10^3$ experienced grade 2–4 toxicity compared with 0/9 with gut TS/β-actin $<5.9 \times 10^3$ (p=0.009). 3/6 responding patients had very high levels of TP compared to 2/11 non-responders, suggesting than unlike 5-FU/LV high tumour TP may be a positive factor for response to RTX. A response was seen in one patient with high DPD. Tumour RTX (polyglutamate) levels were ~28-fold higher than plasma, and were higher in the mucosa of patients experiencing gastrointestinal toxicity (0.15±0.06 ng/mg tissue compared to 0.09±0.06 ng/mg in patients without toxic effects; p=0.09). Plasma dUrd was elevated within 24 h, returned to pretreatment by day 5 in most cases and did not correlate with response or toxicity. These data suggest that individualised therapy, based on gene expression patterns, may be valuable in the treatment of advanced colorectal cancer. In addition, cytotoxicity from RTX may have an underlying molecular cause, and patients at risk could be predicted with reasonable accuracy by pretreatment biopsy.

### 4.6 DIFFERENTIAL EXPRESSION OF DIHYDROPYRIMIDINE DEHYDROGENASE AND THYMIDINE PHOSPHORYLASE IN COLORECTAL TUMOURS AND PRIMARY METASTASES

ESR Collie-Duguid*, SJ Johnston, LM Boyce, J Cassidy and HL McLeod, Dept of Medicine and Therapeutics, University of Aberdeen, Aberdeen AB25 2ZD, UK

Current therapies for colorectal cancer are clearly inadequate as 450 000 people still succumb to the disease each year. Although the aetiology of colorectal cancer is extremely complex, a key feature of the malignant phenotype is uncontrolled cell growth and proliferation. It is essential for actively proliferating cancer cells to increase their rate of DNA synthesis in order to progress through the cell cycle. Increased pyrimidine usage is a common feature in tumour tissues. Thymidine phosphorylase (TP) is the initial enzyme in the salvage pathway of pyrimidine synthesis, thereby regulating levels of pyrimidine nucleosides available for DNA synthesis. Dihydropyrimidine dehydrogenase (DPD) is the initial and rate limiting enzyme of pyrimidine catabolism and results in the degradation of pyrimidine bases, thereby influencing levels of uracil and thymine available for DNA synthesis. Levels of TP and DPD are correlated with the clinical efficacy of pyrimidine anti-metabolites commonly used in the treatment of colorectal cancer. DPD and TP protein levels were measured by Western blotting in primary colorectal carcinomas (n=15), colorectal liver metastases (n=4) and adjacent normal tissues in order to investigate possible differential regulation of these enzymes in colorectal tumours. DPD was reduced in 67% (10/15) of colorectal tumours (mean tumour/normal=0.52, n=10) and in all liver metastases (mean tumour/normal=0.53, n=4) compared to the corresponding normal tissue. In contrast, TP was increased in 80% (12/15) of colorectal tumours (mean tumour/normal=18.91, n=12) and in all metastases (mean tumour/normal=2.61, n=4). TP and DPD protein expression were highly variable in normal (mucosa 474-fold and 45-fold, respectively; liver 4-fold and 2-fold, respectively) and tumour (primary 34-fold and 6-fold, respectively; metastases 2-fold and 3-fold; respectively) tissues. The ratio of TP:DPD was higher in 87% of colorectal tumours and in all liver metastases compared to the adjacent normal tissues. The majority of primary and all secondary colorectal tumours demonstrate co-ordinated down-regulation of DPD and up-regulation of TP protein. This may represent a mechanism whereby the tumour optimises conditions for growth by increasing levels of pyrimidine nucleotides available for DNA synthesis. Further insight into the factors controlling pyrimidine levels in neoplastic tissues will provide novel molecular targets to inhibit DNA synthesis and allow either modulation of existing therapies or development of novel, more effective agents.

### 4.7 CD44V3,8–10 IN PROGRESSION OF COLORECTAL CANCER, AND SERUM LEVELS AS A MEASURE OF TUMOUR EXPRESSION

MG Tutton*[1,2], M George[1,2], S Eccles[2], I Swift[1], AM Abulafr[1], [1]Colorectal Dept, Mayday University Hospital, Surrey, [2]Institute of Cancer Research, Sutton, Surrey, UK

**Introduction** CD44 is an adhesion molecule expressed on epithelial cells with multiple variants derived by alternative splicing. In colorectal cancer, variant 6 has been associated with tumour progression. A newly described variant, CD44v3,8–10, localises with gelatinase B (MMP-9; a key enzyme in invasion and angiogenesis) in breast carcinoma cell invadopodia. We studied colorectal cancers to determine: 1) expression of CD44v3,8–10 in relation to disease progression and 2) whether shed CD44, detectable in sera, reflects expression levels in resected surgical specimens.

**Methods** mRNA was extracted from 13 adenomatous polyps, 50 primary colorectal cancers and paired normal colonic specimens. Using specific primers for CD44std, CD44v3, and CD44v6, RT-PCR products were semi-quantified and correlated both with stage and with preoperative sera levels of the total soluble CD44 by enzyme-linked immunosorbent assay. Band sizes were used to identify specific splice variants.

**Results** CD44v3,8–10 was expressed both within normal colonic tissue and also colorectal cancers with a significant increase both within adenomatous polyps and cancers. This suggests that increases in CD44v3,8–10 are an early event in colorectal cancer, as has been described for variant 6 and confirmed in this study.

| Splice variant | Normal colon | Polyps | Cancers |
|---|---|---|---|
| | Mean expression (relative to β-actin) +/– SE *p<0.05 vs normal | | |
| CD44std | 0.30 +/– 0.09 | 0.08 +/– 0.03 | 0.30 +/– 0.04 |
| CD44v6–10 | 0.98 +/– 0.24 | 1.30 +/– 0.17* | 0.83 +/– 0.14 |
| CD44v8–10 | 0.01 +/– 0.01 | 0.08 +/– 0.06 | 0.12 +/– 0.03* |
| CD44v3,8–10 | 0.46 +/– 0.12 | 1.41 +/– 0.23* | 0.95 +/– 0.10* |

Using CD44std primers the total tumour expression of CD44 was found to significantly correlated with levels of total soluble CD44 in sera (p=0.015; Spearman's correlation). Individual variant analyses will follow where feasible.

**Conclusion** Increased expression of CD44v3,8–10 is an early event in colorectal cancer. This molecule may be involved in tumour invasion via association with MMP-9 and also, via its variant 3 exon (which is a low affinity receptor for growth factors such as bFGF) may potentiate angiogenesis. Changes in tumour expression are also reflected in sera and may be detected by simple serological assays.

### 4.8 COMPARISON OF VASCULAR PERMEABILITY MEASURED BY DYNAMIC MRI AND SERUM/PLASMA VEGF IN LOCALLY ADVANCED RECTAL CANCER

ML George[1,3]*, ASK Dzik-Jurasz[2], A Padhani[2], MO Leach[2], IJ Rowland[2], SA Eccles[3], RI Swift[1], [1]Dept of Surgery, Mayday Hospital, [2]Clinical Magnetic Resonance Research Group, Royal Marsden Hospital, [3]Section of Cancer Therapeutics, Institute of Cancer Research, Sutton, UK

**Aims** Vascular permeability is increased by VEGF and can be assessed radiologically with dynamic MRI. This study examines whether tumour vascular permeability, assessed by paramagnetic contrast enhancement kinetic analysis, correlates with systemic VEGF in locally advanced rectal cancer.

**Methods** 21 patients with locally advanced adenocarcinoma of the rectum, 5 with liver metastasis, were assessed. All examinations were carried out on a 1.5T Siemens Magnetom Vision system using a pelvic phase array coil. A 10 mm slice through the main bulk of the tumour was defined after imaging the full extent of the tumour using a T2 weighted turbo spin echo sequence. Double dose (0.2 mmol $kg^{-1}$) Gd-DTPA was administered at 5 $mls^{-1}$ by power injector and data acquired post contrast for at least 4 minutes. Analysis was performed of line using Tofts model. Serum and plasma VEGF samples were taken from the patients just prior to entering the magnet. VEGF levels were determined by ELISA (R&D Systems, Abingdon, UK). 18 patients had plasma and serum samples and 3 plasma only. Platelet counts were recorded and the VEGF per platelet was calculated.

**Results** Median serum and plasma VEGF were 356.5 $pgml^{-1}$ and 36.9 $pgml^{-1}$ respectively. Whole tumour permeability correlated with serum VEGF ($r = 0.47$, $p = 0.049$) but not plasma VEGF levels ($r = 0.32$, $p = 0.16$). The VEGF/platelet correlated with tumour vascular permeability ($r = 0.53$, $p = 0.025$) but absolute platelet count did not ($r = -0.1$, $p = 0.7$). There was no difference in tumour permeability between those with or without liver metastasis but the numbers are small.

**Conclusion** Only serum VEGF and VEGF/platelet correlated with vascular permeability. The vascular permeability may be affected by VEGF release from platelets within the tumour's microenviroment, with MRI permeability reflecting 'dynamic angiogenesis'.

### 4.9 'IPM': A NEW OUTPATIENT SCHEDULE OF IRINOTECAN, CISPLATIN AND MITOMYCIN FOR THE TREATMENT OF SOLID TUMOURS

J Shamash*, S Slater, J Steele, M Tischkowitz, D Farrugia, P Wilson, C Gallagher, T Oliver, R Rudd, M Slevin, Dept of Medical Oncology, St Bartholomew's Hospital, London ECIA 7BE, UK

**Introduction** The combination of cisplatin and irinotecan shows promise in a wide range of tumors. The optimal schedule remains to be determined and the addition of a third drug may improve efficacy. Mitomycin has proven activity in many solid tumors and was added to make the combination 'IPM'. Cisplatin 40 mg/$m^2$ (days 1 and 15), irinotecan 100 mg/$m^2$ (days 1 and 15 [70 mg/$m^2$ in patients (pts) previously treated with chemotherapy]) and mitomycin C 6 mg/$m^2$ (day 1 only) were administered in a 28-day cycle on an outpatient basis. Toxicity was assessed in five tumours: (1) untreated, inoperable gastric and oesophageal cancer (17 pts, 12 evaluable for response); (2) untreated pancreatic cancer (10 pts, 5 evaluable); (3) relapsed small-cell lung cancer (SCLC) (13 pts, 7 evaluable); (4) cytokine-refractory metastatic renal cell carcinoma (RCC) (15 pts, 14 evaluable); (5) relapsed malignant pleural mesothelioma (7 pts, 4 evaluable).

**Patient characteristics** Median patient age was 54 years (range 35–75 yrs). All pts had performance status 0–3. In the SCLC pts the median progression free interval (PFI) after cisplatin/etoposide-based therapy was 14 months. Four had developed brain metastases despite prophylactic cranial irradiation. The median PFI following cytokine-based therapy in RCC pts was 4 months.

**Toxicity and response** A median of 4 cycles (range 1–6) were administered, of which 143 are evaluable for toxicity. There was one treatment related death. Grade 3–4 toxicities: malaise: 14%; infection: 5%; diarrhoea: 2%; grade 2: alopecia 31%. Objective response rates: (1) untreated gastro-oesophageal: PR 5 (42%), SD 4 (33%), PD 3 (25%); (2) untreated pancreatic cancer. CR 1 (20%), PR 1 (20%), SD 2 (40%), PD 1 (20%); (3) relapsed SCLC: PR 4 (57%) all of whom had brain metastases, SD 2 (29%), PD 1 (14%), median overall survival = 282 days (range 13–377); (4) refractory. RCC: PR 0 (0%), SD 8 (57%), PD 6 (43%), median progression-free survival = 5 months; (5) relapsed malignant pleural mesothelioma: PR 3 (75%), SD 0 (0%), PD 1 (25%).

**Conclusions** IPM is a novel regimen with activity in the 5 tumour types evaluated and has the advantage of outpatient administration with acceptable toxicity.

### 4.10 INHIBITION OF GROWTH OF COLON TUMOUR CELLS BY CURCUMIN CORRELATES WITH INHIBITION OF THE IκB KINASE AND DOWN REGULATION OF CYCLIN D1

Simon Plummer[1], Debbie Wakelin[1], Marianne Bouer[1], Paul Shepherd[1], Lynne Howells[1], Andreas Gescher[1], Sek Chow[2] and Margaret Manson[1], MRC Toxicology Unit[1] and Centre for Mechanisms of Human Toxicity[2], University of Leicester, Leicester LE1 9HN, UK

Curcumin, an antioxidant component of the spice turmeric derived from the rhizome of *Curcuma longa*, possesses a wide range of effects which can be associated with both chemopreventive and anti-cancer activity. We have recently shown that likely targets of curcumin, which account partly for its ability to inhibit induction of the cyclooxygenase-2 (COX-2) gene by colon tumour promoters, are the IκB kinases IKK α and β. Whilst the induction of COX-2 gene expression by tumour promoters is thought to be mediated via AP1 and NF-κB, it has been unclear whether these transcription factors are involved in the constitutive overexpression of COX-2 seen in colon tumour cells. To test the hypothesis that curcumin exerts part of its chemopreventive/anti-cancer effects by inhibiting the growth of tumour cells via inhibition of NF-κB signalling, we examined the effects of curcumin on growth, IKK activity and COX-2 or cyclin D1 expression in the COX-2 overexpressing colon tumour cell line HCA-7. We also examined the effect of the NF-κB inhibitory peptide SN50 and its inactive mutant SN50M on growth. Curcumin inhibited the growth of these cells ($IC_{50}$ ~5μM), without causing a reduction in COX-2 protein levels. SN50 also strongly inhibited growth, but SN50M was inactive. Inhibition of growth by curcumin appeared to involve down-regulation of cyclin D1, and correlated with direct inhibition of IKK ($IC_{50}$ ~5μM). Curcumin's growth inhibitory effects were partly accounted for by induction of cell death which involved chromatin condensation and micronucleation, but was distinguished from classical apoptosis by the lack of DNA laddering.

### 5.1 ACTIVATION OF THE PRO-APOPTOTIC PROTEIN BAK IS A TWO-STEP PROCESS

Bernard Corfe[#], Gareth Griffiths[#], Peter Savory, John Hickman* & Caroline Dive, School of Biological Sciences, G38 Stopford Building, University of Manchester, Manchester M13 9PT UK

We have used conformation-sensitive antibodies raised to two non-overlapping domains of the pro-apoptotic protein Bak in order to investigate cell damage induced changes in this protein. An N-terminal epitope of the pro-apoptotic protein Bak was cryptic in untreated cells but exposed after diverse apoptotic stimuli (etoposide, heat shock, taxol, dexamethasone or staurosporine) in CEM C7A and Jurkat T lymphoma cells[1]. We identified a second conformational change in Bak protein using an antibody raised against the central BH-1 domain. Exposure of this central epitope occurs after the change observed at the N-terminus. Exposure of both the N-terminal and the central epitopes was ZVAD resistant, implying that both either precede, or are independent of, caspase activation. Co-immunoprecipitation studies using a polyclonal antibody to Bak showed that Bak is bound to Bcl-$x_L$ in untreated cells. At early time-points after drug treatment when the N-terminal epitope was exposed but the central epitope remained occluded, Bcl-$x_L$ was bound to Bak. Subsequently, a decrease in binding of Bak to Bcl-$x_L$ correlated with exposure of the central epitope. These data imply that Bak is held bound to Bcl$x_L$ in untreated cells, that exposure of the N-terminus of Bak occurs as a first step in the activation of its pro-apoptotic function, prior to dissociation from Bcl$x_L$. In marked contrast to other members of the Bcl-2 family (Bid, Bax, Bcl-$x_L$ and Bcl-2) where deletion of the N-terminus leads to an increase in lethality, deletion of the Bak N-terminus caused a reduction its ability to kill cells. This finding correlates with the absence of any reported Bak cleavage product during apoptosis and may imply that an intact N-terminus of Bak plays a critical role in integrating damage signals. Taken together these data demonstrate the existence of a two step process in the activation of Bak prior to apoptosis.

1 Griffiths GJ *et al* (1999) *J Cell Biol* **144:** 903–914

[#]These authors contributed equally to this work.
*Current Address: Institut Servier de Recherches, Paris

### 5.2 BAX ISOFORM EXPRESSION IN TUMOUR CELL LINES: LACK OF ASSOCIATION WITH DRUG RESISTANCE

AL Thomas*, SG Martin, J Carmichael, and JC Murray, University of Nottingham Laboratory of Molecular Oncology, CRC Department of Clinical Oncology, City Hospital, Hucknall Road, Nottingham NG5 1PB, UK

The ability of a cell to avoid apoptosis in response to genotoxic stress is of central importance in its progression to a malignant phenotype, and to the acquisition of drug resistance in cancer. In the course of studies to examine the relationship between resistance to apoptosis-inducing chemotherapeutic agents and expression of the pro-apoptotic gene *bax*, we identified two new isoforms, which arise by alternative mRNA splicing (AL Thomas *et al.* (1999) *Cell Death Diff* **6**: 97). One of these isoforms, now referred to as *bax*-ω, contains all six exons found in the predominant form *bax*-α, and in addition retains a partial 49 nucleotide sequence derived from an incompletely-spliced intron 5. This isoform theoretically encodes a 24 kDa protein, lacking a transmembrane domain but containing the critical BH1-3 domains. We were able to express this protein using *in vitro* transcription/translation assays, and to assess its function by transiently over-expressing *bax*-ω in Jurkat cells. The results demonstrate that *bax*-ω induces apoptosis at levels comparable to bax-α. The second isoform, termed *bax*-ε, encodes a polypeptide of ~1 kDa, the biological activity of which remains unclear. We have evaluated the expression of these new isoforms, as well as the better characterised isoforms *bax*-*α* and – δ, in a range of tumour and normal cell lines by RT-PCR, using isoform-specific primers. We found that only the JAR choriocarcinoma cell line consistently expressed all four isoforms *bax*-α, -δ, -ε, and -ω. All tumour cell lines examined, including K562 erythroleukaemia, MCF-7 breast adenocarcinoma, and HL60 promonocytic leukaemia cells expressed the -α and -ω isoforms. Normal fibroblasts and endothelial cells showed a similar pattern. Expression of the *bax*-ε isoform was observed in all these lines apart from K562. Of particular interest were the NCI-460 non-small cell lung cancer cell line and its two etoposide-resistant variants 460 v and 460pV8, since it was in one of these mutant lines that we first detected the new *bax* isoforms. All three lines expressed *bax*-α and -ω, while only 460 v showed expression of *bax*-ε. These data suggest that, in the case of the NCI-460 cell line, resistance to the apoptosis-inducing agent etoposide is not associated with expression of particular *bax* isoforms. The role of these new isoforms of the pro-apoptotic gene *bax*, and of *bax* isoforms in general, remains unclear.

*This work was supported by grants from the Cancer Research Campaign and Eli-Lilly Pharmaceuticals*

### 5.3 RESISTANCE TO TRAIL-INDUCED APOPTOSIS IN BURKITT'S LYMPHOMA

Amalia Mouzakiti* and Graham Packham, CRC Wessex Medical Oncology Unit, Cancer Sciences Division, School of Medicine, University of Southampton, Southampton, SO16 6YD, UK

The TRAIL-R (TNF related apoptosis inducing ligand receptors) DR4 and DR5 are members of the TNF receptor superfamily and are potent inducers of apoptosis in sensitive cells. These death receptors have attracted considerable interest because of their involvement in apoptosis induced by chemotherapeutic agents and radiation, and their potential for direct use in cancer therapies. Although normal cells are thought to be resistant to TRAIL-induced apoptosis, the extent to which individual cancers are sensitive to killing are not known. Potential mechanisms of resistance include mutation/downregulation of DR4/DR5 expression, overexpression of non-functional 'decoy' receptors (DcR1 and DcR2) and altered expression of signalling intermediates (e.g., FADD, caspase-8) or inhibitors of signalling (e.g., FLIP). In this study we investigated sensitivity to TRAIL-induced apoptosis in Burkitt's lymphoma (BL) cell lines.

We characterised the response to recombinant TRAIL of a panel of Epstein Barr Virus (EBV)-positive and negative BL cell lines. Although the majority of lines express the functional TRAIL receptors, DR4 and DR5, only a subset of EBV negative cell lines were highly sensitive to TRAIL-induced apoptosis. SSCP analysis of the intracellular signalling domain of DR4 and DR5 did not reveal potential inactivating mutations in resistant cell lines. Moreover, studies using isogenic BL lines differing only in their EBV status, ruled out a direct role for EBV in determining resistance to TRAIL. p53 and Bcl-2 family proteins are also unlikely to be key determinants of sensitivity in BL cells, as the expression of Bcl-2 family proteins was unrelated to resistance and stabilisation of p53 was not observed following treatment with TRAIL. The expression of other modulators of TRAIL signalling (FADD, caspase-8, FLIP and decoy receptors) did not correlate with responsiveness to TRAIL cytotoxicity. In conclusion, our studies of BL cell lines demonstrate differential sensitivity to TRAIL. However, the molecular determinants underlying TRAIL sensitivity in BL remain to be determined.

### 5.4 EXPRESSION AND FUNCTION OF BCL-2 ASSOCIATED ATHANOGENE-1, BAG-1, IN HUMAN BREAST CANCER CELL LINES

Paul A Townsend* and Graham Packham, CRC Wessex Medical Oncology Unit, Cancer Sciences Division, School of Medicine, University of Southampton, Southampton SO16 6YD, UK

BAG-1 is a multifunctional protein that associates with a range of cellular targets, including the anti-apoptotic BCL-2 protein, the estrogen receptor (ER) and some growth factor receptors. Direct interaction with 70 kDa heat shock proteins (hsp/hsc70) may mediate these diverse associations. BAG-1 alters the in vitro chaperone function of hsp/hsc70 and suppresses apoptosis and regulates steroid hormone dependent transcription. We previously demonstrated that BAG-1 is highly expressed in a subset of invasive breast cancers and identified three distinct BAG-1 isoforms generated by alternate translation of a single BAG-1 mRNA. Translation of the most abundant BAG-1 isoform (p36 BAG-1) initiates at an internal AUG codon, whereas larger BAG-1 proteins (p46 (BAG-1M) and p50 (BAG-1L)) initiate at upstream AUG and CUG codons, respectively. The BAG-1 isoforms are differentially localised and may therefore possess distinct functions. Since apoptosis and the ER are critical determinants of the development and progression of breast cancer, it is important to understand the mechanism of expression and functional role of these individual BAG-1 isoforms.

We characterised the expression of BAG-1 mRNA and protein isoforms in a panel of eight human breast cancer cell lines using RNase protection assays and Western blotting, respectively. We found a discordance between levels of protein and RNA indicating an important level of post-transcriptional control. Using immunoprecipitation of MCF7 cells stably overexpressing p36 BAG-1, we demonstrated a particularly strong interaction with hsc70 relative to binding to BCL-2 and ER. Overexpression of p36 BAG-1 completely suppressed the inhibition of clonogenicity of MCF7 cells following transient heat shock. We are currently constructing a series of BAG-1 mutants and will test the ability of these molecules, as well as the other BAG-1 isoforms, to bind hsc70 and suppress heat shock responses in breast cancer cells. Finally, we are constructing an inducible p36 BAG-1 expression system to investigate directly the effects of BAG-1 on short-term effects of heat shock (e.g., cell cycle arrest, apoptosis). These studies will help us to understand further the role of BAG-1 in breast cancer.

### 5.5 TRANSCRIPTIONAL CONTROL OF BCL-$X_L$ IN MALIGNANT LYMPHOCYTES

Lynn Wood*[1], Lucy MacCarthy-Morrogh[2] and Graham Packham[1], [1]CRC Wessex Medical Oncology Unit, Cancer Sciences Division, School of Medicine, University of Southampton, Southampton, SO16 6YD, [2]Endocrinology and Metabolic Medicine and Sterix Ltd., Imperial College School of Medicine, St. Mary's Hospital, London W2 INY, UK

BCL-$X_L$ is an anti-apoptotic member of the BCL2 family of proteins. It plays a key role in lymphocyte maturation and activation and is widely expressed in lymphomas. Expression of BCL-$X_L$ in lymphoid cells is up regulated at the level of transcription by survival signals such as CD40, IL-2 and IL-3. However the factors involved in transcriptional control of BCL-$X_L$ expression are not fully understood. In this study we investigated the expression of BCL-$X_L$ in lymphoma cells. Using 5′-RACE analysis we identified several BCL-$X_L$ mRNA structures and observed unexpected complexity at the 5′ non-coding portion of BCL-$X_L$ transcripts in human lymphoma cells. Significantly, we identified a novel upstream non-coding exon (1B) spliced directly to exon 2. This novel exon is derived from genomic sequences approximately 1 kb upstream from the previously published non-coding first exon of BCL-$X_L$. A candidate TATA sequence and consensus transcription factor binding sites were located upstream of exon 1B supporting the possible usage of a previously uncharacterised promoter. mRNAs containing a second novel exon (1C) derived from sequences within intron 1 were also identified. To examine cytokine induced regulation of BCL-$X_L$ expression, IL-3 dependent 32D.3 murine myeloid progenitor cells were used in a DEAE-dextran based transient transfection protocol. Luciferase reporter assays demonstrated that IL-3 activation of the BCL-X promoter, following a period of cytokine withdrawal, is dependent on a region 3.2–1.2 kb upstream of the translation initiation site. This potential, murine IL-3 responsive promoter is located within the same genomic region as the novel exon 1B we identified in human lymphoma cells and does not contain the STAT binding sites recently reported to activate BCL-$X_L$ expression. We are currently fine mapping BCL-X promoter elements to identify transcription factors important for BCL-$X_L$ expression in lymphoma cells and in response to survival signals. Our findings suggest that the regulation of BCL-$X_L$ transcription in malignant lymphocytes may differ from that in normal lymphocytes and involve usage of variant exons and promoters. Existence of separate transcriptional control mechanisms for BCL-$X_L$ in malignant lymphocytes potentially could be manipulated in the development of therapies to treat lymphomas.

### 5.6 ASSESSMENT OF MITOCHONDRIAL DNA DAMAGE BY CHEMOTHERAPEUTIC AGENTS

SK Lo, B Tolner, G Rogers, AHV Schapira, JA Hartley and D Hochhauser, Dept of Oncology and Neurology, Royal Free and University College Medical School, UCL, 91 Riding House St., London W1P 8BT, UK

The mitochondrial genome consists of a double-stranded circular molecule of 16.5 kilo bases encoding 37 genes; 2 rRNAs, 22 tRNAs and 13 polypeptides all of which are subunits of enzyme complexes of the oxidative phosphorylation system. It has been reported that cancer cells contain greater numbers of mitochondria and exhibit an increased mitochondrial membrane potential. This could be exploited in targeting tumour cells using charged molecules such as lipophilic cations which show preferential uptake by mitochondria. The rhodocyanine analogue MKT 077 which has been demonstrated to directly damage mitochondrial DNA (mtDNA) is undergoing evaluation as an anticancer drug. The aim of this study was to investigate the effect of chemotherapeutic agents including MKT 077 on mtDNA damage. For these experiments, two cell lines were used; A549 a non small cell lung cancer and its rho 0 derivative which is devoid of mtDNA. Upon exposure to Daunoblastin (an anthracycline) or MKT 077 the rho 0 cell line was found to be approximately 10 fold more resistant to both agents when analysed with a cell proliferation assay (Sulforhodamine B). Immunoblotting demonstrated no difference in p53 or topoisomerase II∝ expression between the cell lines. The involvement of P-glycoprotein was excluded since Daunoblastin uptake was similar in both cell lines as shown by flow cytometry analysis. Southern blot experiments using probes specific for genomic and mitochondrial genes revealed that following treatment with Daunoblastin or MKT 077, there was preferential damage to mtDNA at concentrations of drug in which no nuclear DNA damage was demonstrable. Flow cytometry analysis following treatment with MKT 077 showed a G2 arrest of A549 cells whereas at similar drug concentrations there was no alteration in cell cycle distribution in rho 0 cells. This suggest that mtDNA damage may lead to cell cycle alteration. A fusion cell line was produced by introducing mtDNA from wild type A549 cells into the rho 0 cell line; this restored the chemotherapeutic sensitivity to that comparable with the parental cell line. In summary, we have demonstrated that mtDNA is susceptible to damage by cytotoxic agents and that mtDNA may represent a novel target for chemotherapeutic agents.

### 5.7 PRECLINICAL PHARMACOLOGY OF A NOVEL CLASS OF BCL-2 RESISTANT CHEMOSENSITISER WITH AFFINITY FOR THE MITOCHONDRIAL BENZODIAZEPINE RECEPTOR

[1]DA Fennell, [1]A Okaro, [1]M Corbo, [2]DE Banker, [2]F Applebaum, [1]FE Cotter, [1]St Bartholomew's & The Royal London Medical School, Turner Street, London, UK, [2]The Fred Hutchinson Cancer Research Center, Seattle, USA

Mitochondria play a critical role in regulating commitment to apoptosis, a process underlying the efficacy of most anti-cancer therapies. Resistance mediated at the mitochondrial level by overexpression of Bcl-2 and/or its anti-death homologues limits therapeutic efficacy. We have investigated ligands of the 18 k Dalton mitochondrial benzodiazepine receptor (mBzR) which co-localises the mitochondrial permeability transition pore complex (PTPC), a target for both pro- and anti-death homologues of the Bcl-2 family. The mBzR ligand PK11195 was shown to facilitate chemotherapy induced mitochondrial inner membrane depolarisation (using DiOC6(3)-propidium iodide flow analysis) and apoptosis (Z.DEVD.fmk inhibitable plasma membrane unpacking) in a wide range of haemopoietic and solid tumour cell lines shown to express the mBzR using the fluorochrome, NBD FGIN-1–27 analogue. Intrinsic cytotoxic efficacy was not observed. *Ex-vivo* potentiation of etoposide induced apoptosis was observed in Bcl-2 hyperexpressing lymphoma xenografts after *in vivo* treatment with PK11195 and the mBzR ligand NF49. Furthermore, growth retardation and regression were observed in cholangiocarcinoma (TFK-1, EGI-1) and haemopoietic (BV173) xenografts respectively as early as 72 hours after treatment. At the subcellular level, PK11195 induced rapid mitochondrial biogenesis secondary to induction of reactive oxygen species (ROS) that was inhibitable by antioxiants, but not deletion of the mitochondrial respiratory chain. ROS production was temporally associated with inhibition of glycolytic flux. Cellular levels of reduced non-protein thiols but not Bcl-2 expression, determined cellular sensitivity to PK11195 underlying the importance of its pro-oxidant activity. In summary, mBzR ligands represent a new class of chemosensitiser with clinical potential in the treatment of malignancy.

### 5.8 PK11195, A MITOCHONDRIAL BENZODIAZEPINE RECEPTOR ANTAGONIST RADIOSENSITIZES BCL-$X_L$ AND MCL-1 EXPRESSING CHOLANGIOCARCINOMA TO APOPTOSIS

MC Okaro*[1&2], DA Fennell[2], FE Cotter[2], BR Davidson[1], [1]University Dept Surgery RF & UCMS London NW3 2PF & [2]Dept Exp. Hematology, St. Barts and The RLS of Medicine London E1 2AD, UK

Inoperable cholangiocarcinoma responds poorly to chemotherapy and radiotherapy. DNA damage with the induction of apoptosis following chemotherapy and radiotherapy plays a major part in the mode of action of these forms of cancer treatment. The mitochondria have emerged as key players in apoptosis. Members of the bcl-2 family of apoptosis regulating proteins that localize to the mitochondria play an important role in apoptosis through regulating the release of cytochrome c from the intermembrane space. High levels of bcl-$x_L$ and mcl-1 proteins are localized to mitochondria in cholangiocarcinoma, but their role in the control of apoptosis in this disease remains unknown.

Using Pk11195 a mitochondrial benzodiazepine receptor antagonist that is known to block the cytoprotective effects bcl-2. We investigated the role the highly expressed mitochondrial bcl-$x_L$ and mcl-1 proteins in Egi-1 and Tfk-1 (two human cholangiocarcinoma cell lines) played in radioresistance of cholangiocarcinoma, by studying the effects blocking mitochondrial bcl-$x_L$, and mcl-1 with Pk11195 had on the response of Tfk-1 and Egi-1 to UV Irradiation and 50–1000 cGy of radiotherapy over 72 hrs.

Apoptosis was monitored through measuring the collapse in the inner mitochondrial membrane potential ($\Delta\Psi_m$) detected at single cell resolution by flow cytometry using the $\Delta\Psi_m$ sensitive lipophilic flourochrome $DiOC6_{(3)}$ and by the annexin V assay.

We found that inhibiting mitochondrial bcl-$x_L$ and mcl-1 in cholangiocarcinoma cells sensitizes them to UV light and radiotherapy induced apoptosis which was dose and time dependent. Pk11195 alone had no intrinsic apoptosis inducing effects over 96 hours on either cell line.

Antagonizing the function of the anti-apoptotic bcl-2 proteins at the mitochondria sensitizes cholangiocarcinoma cells to radiotherapy. Expression of these proteins in this disease may explain the low efficacy of radiotherapy in this disease.

### 6.1 MULTIPLE FUNCTIONS OF THE *PTEN* TUMOUR-SUPPRESSOR PROTEIN IN HUMAN PROSTATIC CELL LINES

R Michael Sharrard* and Normal J Maitland, YCR Cancer Research Unit, Department of Biology, University of York, York YO10 5YW, UK

The tumour suppressor protein PTEN has been characterised as a phosphatase with both protein and lipid substrates. Its activities have been predicted to influence cell motility, adhesion and spreading via integrin-activated cellular mechanisms as well as regulating cell proliferation and survival through dephosphorylation of phosphatidylinositol-3,4,5-trisphosphate and related compounds.

Since the PTEN gene is frequently inactivated in prostate cancers, we investigated the function of PTEN phosphatase activities in human non-tumour (PNT2, PNT1a) and tumour (LNCaP, PC3) cell lines using constructs expressing wild-type (wt) PTEN or PTEN mutants G129E (lacking lipid phosphatase), G129R (lacking lipid and protein phosphatases), and C124A (phosphatase-null mutant). We also used Flag-epitope tagged and EGFP-conjugated forms of these variants. Analysis of transiently- and stably-transfected cells by immunocytochemistry and Western blotting demonstrated that PTEN affects cell adhesion and survival through as yet uncharacterised activities additional to both phosphatase activities. Modification of either N- or C-termini of PTEN dramatically altered its stability and function, potentially through effects on interaction with docking or modifying molecules.

Stable overexpression of exogenous wt PTEN was lethal to the LNCaP and PC3 tumour cell lines, which lack endogenous wt PTEN, but not to the non-tumour cell lines, indicating that one or more signalling pathways controlled by PTEN are altered in the tumour cells. The effects of transfection with wt PTEN on the PKB/Akt pathway differed between the cell lines depending on the extent to which Akt phosphorylation could be inhibited solely through blockade of the P13-kinase. Further evidence of differential responses of the downstream targets of PTEN was provided by the effects of both serum starvation and the P13-kinase inhibitor LY294002.

We conclude that PTEN has multiple functions in prostatic cells, including effects mediated through the lipid phosphatase and the PKB/Akt pathway, through the protein phosphatase, and through other, presently uncharacterised functions. The extent to which PTEN affects cell growth and survival through these different pathways may differ between different androgen-dependent and -independent tumour cell lines as well as between tumour and non-tumour cells. Analysis of the functional domain structure of PTEN will allow the design of modified PTEN molecules for targeting specific regulatory pathways implicated in the growth and survival of prostate tumour cells. In combination with suitable delivery systems, the use of PTEN variants with enhanced intracellular stability and specifically modified functions may have important potential in gene therapy for prostate and other cancers.

### 6.2 RNA POLYMERASE III TRANSCRIPTION – ITS CONTROL BY TUMOUR SUPPRESSORS AND ITS DEREGULATION IN CANCERS

S Allison, T Brown, C Cairns, Z Felton-Edkins, C Larminie, A McLees, P Scott, G Sourvinos, T Stein, J Sutcliffe, K Tosh, A Winter and R White*, Institute of Biomedical and Life Sciences, University of Glasgow, Glasgow, UK

The level of protein synthesis is a critical determinant of the rate of cellular growth. Abnormal activation of this process is a frequent feature of transformed and tumour cells. Since translation is restricted if rRNA or tRNA levels are limiting, the synthesis of these products may need to increase to allow the rapid growth of tumours. RNA polymerase III (pol III) is responsible for producing tRNA and 5S rRNA. A wide range of transforming agents stimulate pol III transcription. Our work aims to characterize the mechanisms responsible.

We have shown that the tumour suppressor RB binds and represses the pol III-specific transcription factor TFIIIB (1,2). Many of the molecular details of this regulation are now understood. RB function is lost in cancer through one of three different mechanisms: mutation of the *Rb* gene; binding of oncoproteins; or phosphorylation by cyclin-dependent kinases; we have evidence to show that each of these mechanisms can result in elevated pol III activity in vivo (3).

Release of TFIIIB from repression by RB is likely to contribute to the deregulation of pol III in many tumours. However, additional mechanisms are also involved. For example, TFIIIB is also bound and repressed by wild-type p53 (4); mutations in p53 can release TFIIIB from an important restraint, both in tumours and in untransformed fibroblasts from patients with Li-Fraumeni syndrome. TFIIIB is recruited to promoters by the DNA-binding factor TFIIIC2. TFIIIC2 is a complex of five separate subunits, all of which are overexpressed together in some gynaecological tumours; this provides another route towards the aberrant activation of pol III transcription. Deregulation of pol III is therefore a very common feature of cancer that can involve several distinct regulatory mechanisms.

1 White RJ, Trouche D, Martin K, Jackson SP and Kouzarides T (1996) *Nature* **382:** 88–90
2 Larminie CGC, Cairns CA, Mital R, Martin K, Kouzarides T, Jackson SP and White, RJ (1997) *EMBO J* **16:** 2061–2071
3 Larminie CGC, Sutcliffe JE, Tosh K, Winter AG, Felton-Edkins ZA and White RJ (1999) *Mol Cell Biol* **19:** 4927–4934
4 Cairns CA and White RJ (1998) *EMBO J* **17:** 3112–3123

### 6.3 TRUNCATING MUTATIONS OF THE *EP300* ACETYLASE IN HUMAN CANCERS

Simon A Gayther*[1,3], Paul Russell[1,3], Bruce AJ Ponder[1,3,4], Tony Kouzarides[2,5] and Carlos Caldas[1,4], Departments of Oncology[1] and Pathology[2], and Strangeways Research Laboratories[3], Cambridge Institute for Medical Research[4] and Wellcome/CRC Institute[5], University of Cambridge, Cambridge CB2 2XY, UK

EP300 is a histone acetyltransferase that regulates transcription via chromatin remodelling. It plays an important role in the processes of cell proliferation and differentiation, and interacts with and acetylates p53 in response to DNA damage. This modification regulates the DNA binding and transcription functions of p53. A role for EP300 in cancer has been implied by the fact that it is targeted by viral oncoproteins and that it is fused to MLL in leukaemia. Nevertheless direct demonstration of the role of EP300 in tumorigenesis by inactivating mutations in human cancers has been lacking. Here we describe *EP300* mutations which are predicted to result in a truncated protein in 6 (3%) of 193 epithelial cancers analysed. Of these six mutations, two were in primary tumours (in a colorectal cancer and in a breast cancer), and four were in cancer cell lines (colorectal, breast, and pancreatic). In addition, a somatic in-frame insertion in a primary breast cancer, and missense alterations in a primary colorectal cancer and in two cell lines (breast and pancreatic), were identified. Inactivation of the second allele could be demonstrated in 5 of the 6 cases with truncating mutations, and in 2 of the other cases. Truncated EP300 was shown to be stably expressed in cell lines with EP300 mutations by western blotting. Finally, immunohistochemical analysis suggests that EP300 expression is frequently reduced or absent in breast and ovarian tumours. Together, these data show that *EP300* is mutated in epithelial cancers and for the first time provide unequivocal evidence that it behaves as a classical tumour suppressor gene.

### 6.4 PPARγ EXPRESSION IN THE NORMAL AND NEOPLASTIC HUMAN BLADDER AND PROSTATE AND ITS POTENTIAL AS A NOVEL THERAPEUTIC TARGET

MC Jarvis*, MC Bibby, TA Baokbah, NB Hussain, S Ganta and MJ Thompson, Clinical Oncology Unit, University of Bradford, West Yorkshire BD7 1DP, UK

Prostate cancer is the second most common male cancer in the U.K. Most patients present with androgen dependent tumours, which are treated by the withdrawal of androgen. However many relapse into an androgen independent form, for which currently there is no effective treatment. Bladder cancer is a predominantly male disease. It is initially treated by surgery but has a poor five-year survival rate. Improvements on these figures rely on the development of new adjuvant therapies. The induction of differentiation may offer a novel, alternate approach to the treatment of these types of cancer.

The peroxisome proliferator-activated receptor gamma (PPARγ) belongs to the nuclear receptor superfamily of ligand-activated transcription factors. It has a distinct regulatory role in modulating lipid homeostasis and promoting differentiation. Prostate and bladder tissues were examined for the possible expression of this receptor. Although its function within these, is unknown.

Initially immunostaining was employed to define the distribution of PPARγ within the human urogenital system. Expression of PPARγ was found to be predominantly in the nucleus. Smooth muscle and basal cells of normal prostatic epithelium express the receptor. The majority of androgen responsive (12/13) and androgen unresponsive (7/10) prostate tumours were also shown to stain positively for PPARγ. In the normal bladder the receptor is confined to the nuclei of the urothelium, lamina propria and smooth muscle. 8/9 sections taken from transitional cell carcinomas of the bladder were found to express PPARγ. Western blots on three bladder tumour cells lines, RT4, T24/83 and RT112 confirmed a band at the correct molecular weight for PPARγ (48 kDa). The chemosensitivity of these three bladder tumour cells to troglitazone ($C_{24}H_{27}NO_5S$), a thiazolidinedione agent and selective ligand for PPARγ was measured by the MTT assay. The $IC_{50}$ values for the assay of RT4, T24/83 and RT112 against troglitazone were calculated to be 5.21, 28.74 and 22.35 μM respectively. This indicates that in all lines studied, this specific ligand has a significant effect on cell growth.

The results demonstrate that PPARγ may be a useful, additional therapeutic target for the treatment of some common male urogenital cancers.

This work is supported by War on Cancer and Johnson Matthey by the Kevin Gibbons Award.

### 6.5 ROLE OF E2F-1 IN CHEMOSENSITIVITY TO MINOR GROOVE BINDING AGENTS

C Hampel[1], B Tolner[1], SD Webley[1], E Lam[2], JA Hartley[1] and D Hochhauser[1], [1]Royal Free and University College Medical School, University College London, 91, Riding House Street, London W1P 8BT, [2]Ludwig Institute, St Mary's Hospital London, UK

The E2F transcription factors are critical genes involved in cell cycle progression and have been implicated in apoptosis. Previous studies indicated that cell lines overexpressing E2F-1 exhibited an altered chemosensitivity to chemotherapeutic agents such as 5-fluorouracil, etoposide and doxorubicin (Banerjee et al., 1998 *Cancer Research* **58**, 4292–4296). The aim of the current study was to investigate mechanisms underlying the differential chemosensitivity of a stably transfected E2F-1 overexpressing HT1080 fibrosarcoma cell line to chemotherapeutic agents. Two clones were selected with approximately 3–5 fold increased E2F-1 expression as assessed by Western blotting, compared to the HT1080 control cell line, transfected with the neomycin resistance gene. Electrophoretic mobility shift assay and antibody supershift experiments confirmed increased E2F-1 expression as well as indicating complex formation between E2F-1 and the pocket proteins p130 and p107. No difference in cytotoxicity was observed between the control and E2F-1 overexpressing lines with cisplatinum and melphalan. Growth inhibition studies (sulphorodamine B assay) with BGIII21, a DNA sequence specific minor groove alkylating agent, revealed that E2F-1 overexpressing clones were >8 fold and >70 fold resistant after 1 hour and 72 hour incubation, respectively, compared to the control cell line. The E2F-1 overexpressing cell line was found 3–5 fold less active to other minor groove binding agents such as Hoechst 33258 and anthramycin compared to the control. Clonogenic assays exhibited increased numbers of colonies compared to the control following exposure to BGIII21. Cell cycle analysis and apoptosis assay, at a dose of BGIII21 corresponding to the $IC_{50}$ resulted in induction of cell cycle arrest at the G2 – S phase after 48 hours, followed by apoptotic death in the control cell line. In contrast, cells overexpressing E2F-1 showed neither change in cell cycle distribution nor evidence of apoptosis following drug treatment. Single-strand-ligation-PCR (Sslig) assays are being carried out to investigate whether E2F-1 is implicated in the repair of BGIII21 lesions and whether downstream pathways are affected.

### 6.6 BARX2: A TUMOUR PROGRESSION-SUPPRESSOR AND MODULATOR OF CISPLATIN RESISTANCE IN OVARIAN CANCER?,

H Gabra*[1], G Sellar[1], Li Li[1], K Watt[1], B Nelkin[2], G Rabiasz[1], E Miller[1], D Porteous[3] and JF Smyth[1], [1]ICRF Medical Oncology Unit, Western General Hospital, Edinburgh EH4 2XU, [2]Oncology, Johns Hopkins University School of Medicine, Baltimore MD 21231, [3]Molecular Medicine Centre, University of Edinburgh, Western General, Hospital, Edinburgh EH4 2XU, UK

We have previously reported that frequent loss of heterozygosity (LOH) at 11q24 is associated with poor survival in epithelial ovarian cancer. Microcell mediated chromosome transfer of human chromosome 11 into ovarian cancer cell line OVCAR3 was associated with inhibition of matrigel invasion, transwell migration and cell adhesion.

Human BARX2, initially identified as a ras responsive transcription factor, is homologous to the Drosophila bar class of homeobox genes. We have further refined the location of BARX2, linking it to D11S4131 by GB4 radiation hybrid mapping and physically mapping it to the interval D11S912-GCT17D11.

Northern analysis showed absent BARX2 expression in ovarian cancer cell lines OA W42 and A2780, although genomic PCR showed all exons to be intact. Expression in sequential series of cell lines derived from two patients showed loss of BARX2 expression associated with cisplatin resistance in both patients.

Two mis-sense mutations have been identified in a panel of cancer cell lines. Both are contained within exon 2, 5′ of the homeodomain. The 5′ end of the gene is methylated in a number of cancer cell lines as demonstrated in MspI/HpaII Southern blots, suggesting a possible mechanism for gene silencing.

Transfection of full length BARX2 cDNA into OA W42 confers in-vitro suppression of growth, migration, adhesion to collagen and matrigel invasion. FACS analysis demonstrates that transfection results in S-phase block. However OA W42 clonogenicity is unaltered in colony formation assays. Azacytidine mediated demethylation results in re-expression of BARX2, suggesting that methylation-silencing is a mechanism of inactivation in this cell line.

BARX2 transfection completely reverses acquired cisplatin resistance in PEO1-CDDP, with no such effect on the PEO1 sensitive parent line.

### 6.7 cDNA ISOLATION, CHROMOSOMAL LOCALISATION, AND BIOCHEMICAL CHARACTERISATION OF THE CANDIDATE HUMAN ONCOGENE, *FIBULIN-4*

WM Gallagher*[1], LM Greene[1], SM Strachan[1], MP Ryan[1], V Sierra[2], L Debussche[2], E Conseiller[2], [1]Conway Institute, Department of Pharmacology, University College Dublin, Ireland; [2]Oncology Department, Rhône-Poulenc Rorer, France

Fibulins are an emerging family of extracellular matrix proteins that have been implicated in a variety of biological processes, including tissue remodelling and fibrogenesis. Certain fibulins may influence tumour development through regulation of tumour cell growth, motility, invasion, along with angiogenesis. Fibulin-4, also known as MBP 1, has been shown previously to display both mutant p53-dependent and -independent oncogenic properties[a]. In addition, fibulin-4 provides new insight with respect to the mutant p53 ‘gain of function’ phenotype, a controversial phenomenon that might have important implications for both cancer biology and therapeutic intervention. Mouse fibulin-4 was originally identified by this research group[a]. Here, we describe the cDNA isolation, chromosomal localisation and biochemical characterisation of human fibulin-4. Firstly, we carried out a search for human expressed sequence tags (ESTs) which displayed greater than 85% homology with the mouse *fibulin-4* cDNA sequence. Using information derived from overlapping human ESTs combined with PCR-based cloning, we isolated the entire human *fibulin-4* cDNA. Expression of human *fibulin-4* mRNA, as determined by Northern blot analysis, was shown to be differentially regulated in a tissue-specific manner, e.g. with a high level of expression in heart, ovarian and colon tissues, with very low levels of *fibulin-4* mRNA in the brain and liver. Using the technique of fluorescence *in situ* hybridisation (FISH), the human *fibulin-4* gene was localised to the distal portion of chromosome 11 band q13, which is a genomic region exceptionally rich in genes and disease-related genetic loci. Moreover, portions of 11q13 are frequently amplified in many common tumours, including breast and oesophageal cancer. *In vitro* transcription/translation of *fibulin-4* cDNA produced a recombinant protein of ~54 kDa. Through the additional use of canine microsomal membranes (CMMs) with standard *in vitro* transcription/translation reactions, one can also stimulate sequence-directed co- and post-translational processing events, such as membrane translocation, signal sequence cleavage and N-glycosylation. Using CMMs, we revealed that fibulin-4 protein is most likely inserted into the lumen of the endoplasmic reticulum and is N-glycosylated. These results are in agreement with the presence of a putative signal sequence at the N-terminus of fibulin-4, along with two candidate N-glycoslyation sites located in the centre of the protein. Finally, a pilot study using a panel of nine paired human colon tumour/normal tissue biopsies revealed up-regulation of *fibulin-4* mRNA, as determined by semi-quantitative RT-PCR, in five of these tumours relative to normal tissue obtained from the same patient. This is consistent with a potential role for fibulin-4 in tumourigenesis, as a candidate oncogene.

a Gallagher WM, Argentini M, Sierra V, Bracco L, Debussche L and Conseiller E (1999) *Oncogene* **18:** 3608

### 6.8 CYCLOOXYGENASE-2, PROSTAGLANDIN-$E_2$, MALONDIALDEHYDE AND MALONDIALDEHYDE-DNA ADDUCTS IN HUMAN COLONIC CELLS

RA Sharma*[1], SM Plummer[2], C Leuratti[2], R Singh[2], B Gallacher-Horley[2], LJ Marnett[3], A Gescher[2] and WP Steward[1], [1]Department of Oncology and [2]MRC Toxicology Unit, University of Leicester, Leicester LE1 9HN and [3]Vanderbilt Cancer Center, Nashville, TN 37232, USA

Cyclooxygenase-2 (COX-2), an enzyme pivotal for prostaglandin (PG) biosynthesis, has been implicated in the pathogenesis of colorectal cancer (CRC). Malondialdehyde (MDA), a mutagen produced by lipid peroxidation and during PG synthesis, forms DNA adducts, predominantly with deoxyguanosine ($M_1$-G). Elevated levels of MDA in CRC tissue appear to correlate with levels of PG-$E_2$[1]. Here the hypothesis was tested that COX-2 activity contributes to the development of the malignant phenotype *via* generation of $M_1$-G adducts. $M_1$-G levels, determined by immunoslot blot[2], in non-malignant human colon epithelial (HCEC) cells and malignant colon lines SW48, SW480, HT29 and HCA-7, varied between 77 and 148 adducts per $10^8$ nucleotides. Adduct levels in HCEC cells could be increased by 24-hour incubation with chemically synthesized MDA. Only HCA-7 and HT29 cells expressed COX-2 protein as adjudged by Western blotting. Basal levels of $M_1$-G correlated significantly with those of MDA determined colorimetrically in the four malignant cell types (r=0.98. p<0.001), but neither correlated with expression of COX-2 or PG-$E_2$. Induction of COX-2 expression in HCEC cells by 75 nM phorbol 12-myristate 13-acetate caused an increase in intracellular MDA from 0.38±0.12 to 1.07±0.32 nmol/mg protein after 4 hours, but did not alter $M_1$-G adduct levels after 4, 24 or 72 hours. Selective inhibition of COX-2 activity in HCA-7 cells by incubation with 17.7 µM NS-398 for 24 hours did not alter MDA nor $M_1$-G levels, despite decreasing PG-$E_2$ levels from 10.7±2.1 to 1.3±0.6 ng/$10^6$ cells. These results do not support a causal link between COX-2 activity and $M_1$-G adduct levels in the mechanism of CRC development. They represent independently regulated variables which merit separate investigation as potential biomarkers of efficacy in chemoprevention trials.

1 Hendickse CW et al (1994) *Brit J Surg* **81:** 1219
2 Leuratti C et al (1998) *Carcinogenesis* **19:** 1919

### 7.1 ASSOCIATION BETWEEN POLYMORPHISMS OF THE *GPX1* GENE AND MULTIPLE PRIMARY TUMOURS OF THE HEAD AND NECK

S Jefferies*[1], Z Kote-Jarai[1], R Houlston[1], M-J Frazer–Williams[1], R A’Hern[2], MPT Collaborators and R Eeles[1]

Head and neck cancer (SCCHN) is a common cancer. For many years it has been known that the major aetiological factors are tobacco and alcohol exposure and little attention has been paid to other predisposing factors. There is now some preliminary evidence that there may be a genetic component to SCCHN. Individuals who have had an index squamous cell cancer of the head and neck (SCCHN) have a 10–20% chance of developing a second primary tumour (MPT). There is an increased risk of cancer in first-degree relatives of individuals with SCCHN and this risk rises markedly when the affected individual has had two primary tumours. Individual susceptibility to cancer development may be contributed to by differences in metabolism of carcinogens. The human cellular glutathione peroxidase I (*GPX1*) is a selenium-dependent enzyme that protects against oxidative damage. Dietary selenium has been implicated in the possible chemoprevention of some cancers. The *GPX1* gene maps to chromosome 3p21, an area frequently showing loss of heterozygosity in SCCHN. There are three *GPX1* alleles characterized by number of GCG triplet nucleotide repeats encoding for alanine in a polyalanine tract in exon 1. The *GPX1* alleles with five or seven alanines are identical except for the length of the polyalanine polymorphism. The *GPX1* ALA6* also has a leucine for proline substitution at codon 198, a T for C substitution at +2 and a G for A substitution at –592[1]. The effect on function of each of these polymorphisms of the *GPX1* gene is not known.

We investigated the association between the genetic polymorphisms in the *GPX1* gene and patients with index squamous cell cancer of the head and neck who developed second primary tumours compared with population controls. The genotypes were determined for 63 cases of MPT and 259 controls using a PCR technique with a fluorescent-labeled primer. The PCR products were analysed using an AB1 automated fluorescent DNA sequencer. The associations between specific genotypes and the development of MPT were examined by the Chi-squared test.

We found a significantly increased frequency of the *GPX1*ALA6/GPX1*ALA7* genotype in the MPT cases versus controls (P=0.05). The findings of this study suggest that the *GPX1*ALA6/GPX1*ALA7* genotype may be associated with an increased risk of developing second primary cancers. The identification of such genotypic markers may be of value in the future for targeting chemoprevention towards individuals at high risk of developing a second malignancy.

MPT Collaborators: J Henk[2], M Gore[2], P Rhys-Evans[2], D Archer[2], K Bishop[2], E Solomon[3], S Hodgson[3], M McGurk[3], J Hibbert[3], M O’Connell[3], M Saunders[4], M Partridge[3], E Chevretton[3], F Calman[3], K Shotton[5], A Brown[6], S Whittaker[7], D Goldgar[8], W Foulkes[9]. [1]Cancer Genetics, Institute of Cancer Research, 15 Cotswold Rd, Sutton, Surrey. [2]The Royal Marsden Hospital Trust, Downs Rd, Sutton, Surrey, SM2 5PT. [3] Guys and St Thomas’ and Kings Hospital Trusts. [4]Mount Vernon Hospital Trust, Rickmansworth Road, Northwood, Middlesex, HA6 2RN. [5]Mid-Kent Oncology Centre, Hermitage Lane, Maidstone, Kent, ME16 9QQ. [6]Queen Victoria Hospital, East Grinstead. [7]Royal Surrey County Hospital, Egerton Road, Guildford, GU2 5XX. [8]IARC, Lyon, France. [9]McGill University, Montreal, Canada.

1 Moscow et al (1994) *Carcinogenesis* **15:** 2769–2773

### 7.2 *BRCA*1, *BRCA*2 MUTATION AND PEDIGREE GENETIC ANALYSIS TO DETERMINE GENETIC RISK IN THE UK ROYAL MARSDEN HOSPITAL TAMOXIFEN PREVENTION TRIAL

RA Eeles[1,2], TP Powles[2], S Ashley[2], DF Easton[3], L Assersohn[2], N Sodha[2], M Dowsett[1], B Gusterson[1], A Tidy[2], G Mitchell[1,2], Z Kote-Jarai[1], 1) Institute of Cancer Research, Sutton, Surrey, SM2 5NG, UK; 2) Royal Marsden NHS Trust, Sutton, Surrey SM2 5PT, UK; 3) CRC Genetic Epidemiology Unit, Strangeways Research Laboratories, Cambridge, CN1 4RN, UK

In the Royal Marsden Hospital tamoxifen prevention study, 2500 women at increased risk of developing breast cancer because of a family history of the disease were randomised to receive tamoxifen 20 mg daily or placebo for 8 years. 70 women developed primary breast cancer, 36 whilst on placebo, 34 on tamoxifen. Family history out to at least 2nd degree relatives was taken from all women in the study. DNA from peripheral blood from 67 of the 70 women was analysed for coding mutations in the *BRCA*1 and *BRCA*2 genes by CSGE analysis of the entire coding region of both genes. 6 mutations were found, 2 in *BRCA*1 and 4 in *BRCA*2, 4 would be expected to be pathogenic as these were nonsense/frameshifts. 2 were rare variants which were not present in 100 normal controls. The posterior probability of carrying a breast cancer predisposition gene in the individuals who developed breast cancer was assessed using the Cyrillic genetic risk package, based on the Claus model (Claus et al., 1991*). 26 women had <50% posterior probability of harbouring a breast cancer predisposition gene and 44 had a ≥50% chance of having a breast cancer predisposition gene. In the former group of 26 women, 8 had been taking tamoxifen and 18 placebo. In the group of women with ≥50% probability of having a breast cancer gene, 26 had been taking tamoxifen and 18 placebo. The differences between the numbers of women taking tamoxifen who subsequently developed cancer in the two groups divided by <50% or ≥50% genetic risk was significant at p = 0.04. These preliminary data suggest that tamoxifen prevention maybe more effective in women with a <50% chance of harbouring a breast cancer predisposition gene.

*Claus et al (1991) *Am J Epidemiol* **48**

### 7.3 BRCA2 MUTATIONS IN A SCOTTISH MALE BREAST CANCER POPULATION

IE Young*[1], MAF MacKenzie[1], KM Kurian[1], C Annink[1], JMV Back[1], IH Kunkler[2], BB Cohen[3], CM Steel[3], 1/C.R.C. Laboratories, Molecular Medicine Centre & 2/Dept. of Clinical Oncology, Western General Hospital, Crewe Road, Edinburgh, EH4 2XU, 3/School of Biomedical Sciences, University of St. Andrews, St. Andrews, Fife KY16 9TS, UK

Male breast cancer is a rare disease, accounting for approximately 1% of total cases of breast cancer. Mutations of the BRCA1 and BRCA2 tumour suppressor genes have been identified in some cases of familial and early onset breast cancer. Male breast cancer has been linked to mutations of the BRCA2 gene in some cases. The aims of this study were to construct family pedigrees and to identify mutations in the BRCA2 gene in a group of male breast cancer patients from the South East of Scotland.

Cases were a consecutive series of 76 male breast cancer patients treated in S.E. Scotland between 1974 and 1998. 64 DNA samples were available (from blood samples in 25 cases, archival tissue in 39). Family pedigrees were constructed for all cases where possible by an experienced genealogist.

The entire coding region and splicing sites of BRCA2 were amplified by PCR using 62 pairs of primers (designed by Dr. R. Wooster). These PCR products then underwent heteroduplex analysis (HA). Protein truncation testing (PTT) was also performed for exons 10, 11 and 27. PCR products showing variant bands on HA or PTT were reamplified from genomic DNA and cycle sequenced.

Germline BRCA2 mutations were identified in 12 of the 64 (19%) male breast cancer cases. This frequency is higher than that previously reported from the UK (7%)[1] or USA (4–14%)[2][3], suggesting that germline BRCA2 mutations may account for a relatively high proportion of male breast cancer cases in Scotland. Six of the 12 cases (50%) had a history of female breast cancer in a first- or second-degree relative. 38 of the 61 male breast cancer patients tested (62%) carry a polymorphism in BRCA2 exon 2 (203 G→A). 52 of 116 male and female controls (45%) carry this polymorphism. The difference between cases and controls is statistically significant (p=0.0272, $\chi^2$=4.88, 1 df). The odds ratio for the risk of a male with the exon 2 polymorphism developing breast cancer is calculated as 2.03 (95% CI 1.08–3.83). Previous studies have found the frequency of the exon 2 polymorphism to be only 17–22% in control and male breast cancer populations. All three male breast cancer cases found to have a mutation in exon 16 (7830 del 16 bp, the commonest mutation found in this study) also carry the exon 2 polymorphism, perhaps indicating linkage disequilibrium. Alternatively, the exon 2 polymorphism may in some way alter the transcription of the BRCA2 gene. This would be an interesting area that we intend to explore further because it may provide further insight into how BRCA2 is involved with the development of male and female breast cancer.

1 Mavraki et al (1997) *Br J Cancer* **76:**(11) 1428
2 Friedman et al (1997) *Am J Hum Genet* **60:** 313
3 Couch et al (1996) *Nat Genet* **13:** 123

### 7.4 ABSENCE OF *BRCA2* CAUSES GENOMIC INSTABILITY

A Tutt, A Gabriel, D Bertwistle, F Connor, H Paterson, J Peacock, G Ross and A Ashworth, Institute of Cancer Research, London SW3 6JB, UK

**Background** Women heterozygous for mutations in the breast cancer susceptibility gene *BRCA2* have a highly elevated risk of developing breast cancer. *BRCA2* encodes a large protein; and although functions in DNA repair and transcription have been suggested, its role in breast carcinogenesis is not clear. We have previously constructed a hypomorphic mutation (*Brca2*$^{Tr2014}$)*(1)*. Embryonic fibroblasts (MEFs) derived from mice homozygous for this mutation or a similar mutation (*Brca2*$^{Tr}$)(2) proliferate poorly in culture, and overexpress p53 and p21$^{Waf1/Cip1}$. These MEFs have intact p53-dependent DNA damage $G_1$/S(1, 2) and $G_2$/M checkpoints(2), impairment of DNA double strand break (dsb) repair(1) and develop chromosome aberrations(2).

**Aim of study** To further examine the role of *Brca2* in the maintenance of genomic stability at the chromosomal level, using spontaneous induction of micronuclei and examination of chromosome segregation.

**Methods** MEFs at passage 2 or 3 were fixed, stained with DAPI, and at least 200 MEFs per data point were sampled from each of 3 embryos per genotype (*Brca2* +/+; *Brca2*$^{Tr2014/+}$, *Brca2*$^{Tr2014/Tr2014}$) in 3 independent experiments to score micronucleus frequency and centrosome number. Micronucleus type was scored by FISH using a pancentromeric probe The centrosome (mitotic spindle organising centre) was labelled with an anti-γ tubulin antibody and detected by immunofluorescent microscopy. Metaphase chromosome spreads were made using standard methods.

**Results** Homozygous mutation in *Brca2* leads to the formation of spontaneous micronuclei, a marker of chromosome instability. The proportion of cells containing micronuclei increased with passage. Chromosomal missegregation was a major mechanism leading to formation of micronuclei, and resulted in significant aneuploidy. *Brca2*$^{Tr2014/Tr2014}$ *MEFs* developed centrosome amplification at low passage, and such cells also contained micronuclei.

**Conclusions** We have demonstrated a role for Brca2 in maintenance of genomic stability at the chromosomal level. These data suggest a mechanism whereby loss of *Brca2* may drive the loss of cell cycle regulation genes thus enabling proliferation and tumorigenesis.

1 Connor F, Bertwistle D, Mee PJ, Ross GM, Swift S, Grigorieva E, et al (1997) Tumorigenesis and a DNA repair defect in mice with a truncating Brca2 mutation. *Nat Genet* **17**:(4) 423–30
2 Patel KJ, Vu VP, Lee H, Corcoran A, Thistlethwaite FC, Evans MJ, et al (1998) Involvement of Brca2 in DNA repair. *Mol Cell* **1**:(3) 347–57

### 7.5 DIFFERENCES IN THE BIOLOGICAL AND CLINICAL EFFECTS OF VOROZOLE AND TAMOXIFEN IN POST-MENOPAUSAL PRIMARY BREAST CANCER

C Harper-Wynne*[1], K Shenton[1], R Ahern[1], F MacNeill[2], P Sauven[2], I Laidlaw[2], Z Rayter[2], S Miall[2], N Sacks[2] and M Dowsett[1], [1]The Academic Department of Biochemistry, The Royal Marsden Hospital, London SW3 6JJ, [2]Vorozole Study Group, UK

We report serum and tumour-biomarker data from the first randomised study of tamoxifen (T) versus an aromatase inhibitor (AI), vorozole (V), as primary therapy for breast cancer. This was a single-blind multicentre study recruiting postmenopausal patients with ER+ve breast tumours >2 cm diam. 53 patients were randomised, 27 to T 20 mg/day and 26 to V 2.5 mg/day orally for 12 weeks. Serum markers of bone resorption, measured by ELISA, decreased in the T arm after 12 weeks (p=0.006), but did not significantly change in the V group. Tumour biopsies were performed pretreatment (pre) and at 2 and 12 weeks (2 w, 12 w) for analysis of proliferation (Ki67), apoptosis (TUNEL) and ER/PgR status. Ki67 levels decreased between pre & 2 weeks by 58% (mean) with V (p=0.002) and by 43% with T (p=0.04): p=ns for the difference between T & V. There were falls in Ki67 between pre & 12 w by 73% with V (p<0.001) and by 57% with T (p=0.015), with a significant fall between 2 w & 12 w with T (P=0.03). The data indicate a more rapid effect on proliferation with the AI but the later fall with T (2 w–12 w) resulted in no significant difference in Ki67 reduction between the 2 groups over the 12 weeks. Positive correlations, of borderline significance, existed between pre-2 w Ki67 reductions and pre-12 w tumour volume reductions (T; p=0.08 & V; p=0.09). There was a significant decrease in apoptosis between pre & 2 w in the V group (p=0.03) but with no change in the T group. Ki67/apoptosis ratio was reduced at 2 w by 48% (mean) with T (p=0.01) and by 35% with V (ns) and at 12 w by 63% (p=0.01) and 69% (p=0.01) respectively. Serum IGF-1, a recognised anti-apoptotic protein, was non-significantly elevated over 12 wk in the V group and significantly reduced in the T group (p=0.01).

**Conclusion** This study indicates that V does not significantly increase bone loss in this group over 12 weeks. The trend towards proliferation reduction at 2 w predicting volume reduction at 12 w for V supports the use of Ki67 changes at 2 w as an intermediate endpoint. Pharmacokinetic differences between drugs, however, may influence optimal timing of a biopsy to accurately predict for response. The Ki67/apoptosis ratios were similar for both drugs, cytostatic effects predominating in determining the clinical response. It is possible that the difference in the change in apoptosis between the drugs relates to the different effects of T & V on IGF-1. Studies of down stream components of this pathway as well as of bcl-2 family members are therefore being evaluated in these samples.

### 7.6 A PHASE I PHARMACOKINETIC AND PHARMACODYNAMIC TRIAL OF THE VEGF INHIBITOR SU5416 INCORPORATING QUANTITATIVE CONTRAST ENHANCED MR ASSESSMENT OF VASCULAR PERMEABILITY

A O'Donnell[1,2], J Trigo[1,2], U Banerji[1,2], F Raynaud[2], A Padhani[1,2], A Hannah[3], A Hardcastle[2], W Aherne[2], P Workman[2] and I Judson[1,2], 1. Royal Marsden Hospital, Sutton, 2. Institute of Cancer Research, Sutton, 3. SUGEN Inc. Sth San Francisco, USA

SU5416 (Z-3-[2,4-dimethylpyrrol-5-yl)methylidenyl]-2-indolinone) is a potent inhibitor of VEGF mediated Flk1/Kdr receptor signalling. When used against a panel of murine and human tumour cell lines in xenograft models SU5416 demonstrates broad anti-tumour activity with reduction in total and functional tumour vascularization and well as tumour growth inhibition. This Phase I trial investigates traditional endpoints – toxicity and pharmacokinetics but also incorporates pharmacodynamic endpoints – tumour vascular permeability using dynamic contrast enhanced MR and serial VEGF levels in an attempt to provide non-invasive means of determining early biological evidence of effect for an agent which is potentially cytostatic. Sequential cohorts of 3 patients have received escalating doses of SU5416 twice weekly for 4 weeks per cycle. Currently 22 patients (11 M: 11 F) with a variety of tumour types, median age 48 years (20–74 years) have received 43 cycles of SU5416 at doses: 48 mg/m² (3 pts), 65 (3), 85 (3), 110 (3), 145 (3), 145 → 190 (7). No dose limiting toxicity has been observed. Local venous thrombophlebitis is common. Despite premedication, hypersensitivity reactions (attributed to the diluent Cremophor®) requiring additional steroid administration, have occurred in 8 patients however treatment has been able to be continued in each case. All other toxicity was mild. Headache and emesis appear dose related. No haematologic or metabolic toxicity has been observed. Pharmacokinetic studies show rapid drug clearance (mean 74.3 L/hr ± SD 29.5) with the formation of two metabolites – 5′ hydroxyl SU5416 and 5′ carboxyl SU5416. An increase in drug clearance with repeated administration is seen. This has enabled intra-patient escalation in therapy from 145 → 190 mg/m² (190 mg/m² reported as DLT, Rosen ASCO 1999 Abs 618) after 4 doses with no additional toxicity. No tumour responses have been seen however 7 patients have has stable disease with 6 remaining on study for ≥ 12 weeks. Using Magnevist® enhanced MR performed 1–4 hours post dose, a decrease in permeability was seen in 4/6 patients with stable disease. (Rg 12–37%) Conversely in 6/12 patients with progressive disease an increase in vascular permeability was seen. (Rg 17–41%) These changes were observed even at the lowest dose level. VEGF levels in plasma, serum and platelet depleted plasma in patients receiving the final dose level will be correlated with these results. Accrual is ongoing.

### 7.7 A PHASE I STUDY OF A 5-DAY SCHEDULE OF IV TOPOTECAN (T) AND ETOPOSIDE (E) IN UNTREATED SMALL CELL LUNG CANCER (SCLC)

Penny Sutton*[1], Peter I Clark[1], David B Smith[1], Ernest Marshall[1], Kate Hannigan[1] and Graham Ross[2], [1]Clatterbridge Centre for Oncology, Merseyside, UK and [2]SmithKline Beecham Pharmaceuticals, UK

The topoisomerase I inhibitor T and the topoisomerase II inhibitor E are both active in SCLC. The importance of sequence has not been elucidated in cancer patients (pts). A phase I trial explored a schedule combining an intravenous (IV) 5-day regime of both T and E in previously untreated pts with SCLC. T was given as a 30 min infusion, followed by a 30 min saline flush and then a 1 hour infusion of a fixed daily dose of E (60 mg/m²/day) every 3 weeks for a maximum of 6 cycles. Dose levels of T commenced at 0.5 mg/m²/day and were escalated by 0.25 mg/m²/day per cohort. Dose limiting toxicity was defined as 33% of pts with > Grade 3 non-haematological toxicity and/or absolute neutrophil count nadir $<0.5\times10^9$/l for 7 days or complicated by fever requiring IV antibiotics, and/or platelets $<25\times10^9$/l. To date 19 pts have received 105 courses. Median age of pts was 68 yrs; 15 had WHO performance score (PS) 1 and 4 had PS 2; 13 had extensive disease, 6 limited disease. Seven pts were treated in the first cohort of T 0.5 mg/m²/day (41 courses): grade 4 neutropenia was seen in 7 courses (4 pts), grade 3 neutropenia in 11 courses (5 pts) and there were 11 dose delays in 5 patients. One episode of DLT occurred. An initial 6 pts were treated in the second cohort of T 0.75 m/m²/day and 2 episodes of DLT occurred. As one of these occurred in a patient who subsequently had similar toxicity following treatment with dose-reduced CAV, an additional 6 patients were enrolled. 12 pts have received 70 courses of T 0.75 mg/m²/day; grade 4 neutropenia was seen in 24 cycles (10 pts), grade 3 neutropenia in 26 cycles (11 pts) and there have been 19 dose delays in 8 pts. 3 episodes of DLT have occurred. There have been no toxic deaths and apart from hair loss, non-haematological toxicity was <grade 2. All 19 pts are evaluable for response and 18 have responded, 15 PRs and 3 CRs. The median duration of survival is encouraging at 9+ months.

A 5-day schedule of the IV combination of T and E given on the same day is well tolerated and appears very active in pts with untreated SCLC. The recommended phase II dose of a 5-day schedule of this IV combination is T 0.75 mg/m²/day and E 60 mg/m²/day. A phase I study combining the oral formulations of T and E in this 5-day schedule is underway.

### 7.8 A HIGHLY ACTIVE REGIMEN FOR MALIGNANT MESOTHELIOMA WITH NEW BIOLOGICAL DATA

Mendes R[a,b]*, O'Brien MER[a,c], Bromelow KV[b], Gregory KR[a], Norton A[a], Padhani A[a], Smith IE[a], Souberbielle BE[a,b], [a]The Royal Marsden Hospital, Sutton, [b]Molecular Medicine, King's College, [c]Kent Cancer Center, Maidstone, UK

**Background** Intradermal SRL172 (heat-killed *Mycobacterium vaccae*) and chemotherapy have been used in lung cancer and mesothelioma patients with promising results. The rational behind the addition of intratumoral SRL172, in combination with intradermal SRL172 and chemotherapy (mitomycin, cisplatin and vinblastine), is to provoke a pro-inflammatory microenvironment in the tumour which may be immunosuppressive.

**Methods** Fifteen patients were recruited into the study in 4 cohorts. Patients were given intratumoral SRL172 at 3 weekly intervals with chemotherapy. The intratumoral dose of SRL172 was increased 10 fold with each cohort starting at 1 μg up to 1 mg. Intradermal SRL172 (1 mg) was given four weekly to all patients. Patients were monitored for side-effects, clinical and symptomatic response. Tumour assessments were done by CT scan after every 2 courses of chemotherapy. Blood was taken from patients, their spouses and their age-sex matched controls for baseline immunological parameters (lymphocytes markers, T cell functional assays (MLR, mitogenic and recall antigen stimulations) and intracellular cytokine assays for both T cells and NK cells (IL-2, IL-4, γ-INF and TNF-α). At the end of treatment, immunological assessment were repeated on the patients.

**Results** Baseline haemato-immunology showed that the patients, compared to the spouses and the age-sex controls, had lower haemoglobin levels (12.84, 13.43, 14.4 g/dl respectively, p=0.006), high platelet count (mean 383, 237, 227 10⁹/L; p<0.001),), high white cell count (8.96, 6.46, 7.06 10⁹/L; p <0.05), low lymphocyte count (1.3, 1.98, 2.1 10⁹/L; p <0.01), low CD3+ lymphocyte count (1.03, 1.45, 1.52 10⁹/L; p = 0.007), low CD3-ve lymphocyte count (0.36, 0.52, 0.61 10⁹/L; p=0.005), low CD4+CD3+ cell count (0.68, 0.89, 0.9710⁹/L; p<0.05), low NK cell count (0.13, 0.16 0.268 10⁹/L; p=0.04) but higher proportion of activated NK cells expressing CD69 (8.9%, 6%, 3.3%; p <0.05). Cytokine production (IL-2, IL-4, gamma-interferon and TNF-α for T cells, NK-T cells and NK cells) following stimulation with PMA/ionomycin were similar between the patients, spouses and controls. Likewise, whole blood functional assays (mitogenic, allogeneic and recall antigen stimulation) were also similar between these 3 groups. The overall objective response rate was 40% (6/15 PR) although the first 3 cohorts who received 1 μg to 100 μg bacilli had a response rate of 66% (6/9) suggesting that lower intratumoral doses are more effective. Overall symptomatic response was recorded in 57% (8/14) assessable patients. The overall median survival is 12 months. Immunological assessments after treatment revealed that the clinical response correlated with a decrease in platelet count. All patients appeared to have a decrease in IL-4 producing CD3+ve lymphocytes and an increase in the proportion of activated NK cells but these 2 parameters did not correlate with response suggesting that they are non-specific consequences of treatment.

**Conclusion** SRL172 can safely be given intratumorally and we have established the intratumoral dose that is appropriate for future studies. The regimen had high response rate and predictable toxicity profile. In addition, the simple correlation of the platelet count with clinical response should be further investigated in mesothelioma.

We thank SR Pharma for their support.

### 8.1 INTENSITY-MODULATED RADIOTHERAPY (IMRT) FOR TUMOURS OF THE HEAD AND NECK, PELVIS AND THORAX: PRE-CLINICAL EVALUATION AND IMPLEMENTATION

C Nutting*, D Convery, V Cosgrove, C Rowbottom, S Webb, and D Dearnaley, Institute of Cancer Research and Royal Marsden NHS Trust, Surrey SM2 5PT, UK

**Purpose** To evaluate the potential benefits of IMRT compared to current radiotherapy techniques, and to implement clinical trials of IMRT for appropriate tumour sites.

**Methods** 30 patients with head and neck, pelvic and thoracic tumours underwent treatment planning for conventional radiotherapy (RT), 3-dimensional conformal RT (3DCRT) and inverse-planned IMRT. Dose distributions were compared using dose-volume histograms for tumour and normal tissues, and normal tissue complication probabilities were calculated. Methods were developed to optimise beam number and direction to determine the most efficient delivery techniques, and for pelvic tumours a clinical dose escalation trial protocol was designed.

**Results** IMRT treatment plans for thyroid carcinoma and pelvic lymph nodes (tumours with a concave PTV) showed the greatest improvements compared to conventional and 3DCRT. There was 12% reduction in maximum spinal cord dose (p<0.01), and a 70% reduction in pelvic small bowel treated above 45 Gy (p<0.01) respectively. PTV dose homogeneity was improved, and other normal tissues also spared. Oesophageal, parotid, and para-nasal sinus tumours (with moderate or no concavities in the PTV), showed statistically significant but smaller improvements in normal tissue sparing of lungs, oral cavity and cochlea, and optic nerves respectively. 9, 7, or 5 equispaced IMRT fields gave similar benefits, but dose distributions deteriorated with 3 equispaced fields. A computerised optimisation algorithm was designed which, for selected tumour sites, customised the IMRT beam directions allowing both coplanar and non-coplanar beam arrangements. This produced novel techniques that maintained the advantages of multi-field IMRT but using only 3–4 beams. This should reduce the time required for IMRT delivery, and verification. Treatment plans were delivered to humanoid phantoms using a dynamic multi-leaf collimator technique, and the delivered doses were found to be accurate to within 1–2% using photographic film and BANG gels. A Phase 1 clinical protocol was designed to evaluate dose escalated IMRT (50–65 Gy) to pelvic lymph nodes while sparing small bowel. The main endpoints will be clinician and patient assessments of acute and late toxicity, recruitment starting in April 2000.

**Conclusions** IMRT represents a significant advance in conformal radiotherapy. The benefits are greatest for tumours with a concave PTV where normal tissue structures within the concavity can be spared. For non-concave tumours, dose homogeneity is improved compared to current techniques, and for all tumour sites some normal tissue sparing was observed. Treatment delivery is possible with 3–5 optimised beam directions, and clinical assessment of this technique is underway.

### 8.2 PALLIATIVE RADIOTHERAPY FOR MUSCLE INVASIVE BLADDER CANCER: FINAL RESULTS OF A PROSPECTIVE RANDOMISED TRIAL OF TWO RADIOTHERAPY SCHEDULES

JD Graham*, GO Griffiths, BM Uscinska & GM Duchesne, On behalf of the MRC Bladder Cancer Working Party MRC Clinical Trials Unit, 222 Euston Road, London NW1 2DA, UK

**Methods** A multi-centre randomised trial was conducted comparing the efficacy and toxicity of two radiotherapy schedules (35 Gy in 10 fractions and 21 Gy in 3 fractions) for symptomatic improvement in patients considered unsuitable for curative treatment through disease stage or co-morbidity. The primary outcome measures included overall symptomatic improvement of bladder-related symptoms at 3 months and changes in bladder and bowel related symptoms from pre-treatment to end of treatment and 3 month assessments. Overall symptomatic improvement was defined as the improvement in one-clinician-reported bladder-related symptom of at least one grade at 3 months, with no deterioration in any other bladder-related symptom.

**Results** 500 patients were recruited but data on symptomatic improvement at 3 months was only available on 272 patients. Of these 68% (184/272) achieved symptomatic relief, 71% (95/133) for 35 Gy and 64% (89/139) for 21 Gy, a difference of 7% 95% CI (–2%, 13%), p=0.192. There was no evidence of a difference in efficacy or toxicity between the two schedules. There was also no evidence of a difference in survival between the two schedules (HR=0.99, 95% CI 0.82, 1.21, p=0.933).

**Conclusion** This is the largest prospective trial to date in the palliative treatment of bladder cancer and provides baseline data against which other results may be compared. The use of 21 Gy in 3 fractions appears as effective as 35 Gy in 10 fractions, although modest differences in survival, symptomatic improvement rates and toxicity cannot be reliably excluded.

### 8.3 BCL-2 EXPRESSION IDENTIFIES PATIENTS WITH ADVANCED BLADDER CANCER TREATED BY RADIOTHERAPY WHO BENEFIT FROM NEOADJUVANT CHEMOTHERAPY

PW Cooke*[1], R Ganesan[2], A Burton[1], LS Young, DMA Wallace[2], ND James[1], 1. CRC Institute for Cancer Studies, University of Birmingham, 2. Queen Elizabeth Hospital, Birmingham, UK

**Objective** To assess the prognostic significance of Bcl-2 expression on the clinical outcome after radiotherapy for muscle-invasive bladder cancer, and to determine if it is possible to identify a subgroup of patients to whom neoadjuvant chemotherapy can be targeted to improve survival.

**Materials and Methods** Immunohistochemical staining for Bcl-2 and p53 was performed on the tumours of 51 patients with stage T2–T4a NX M0 TCC of the bladder who had been included in a randomised clinical trial of radiotherapy with or without neoadjuvant cisplatin. The association between positive staining and salvage cystectomy rate and overall survival was examined, with a median follow-up of 12 years.

**Results** Positive Bcl-2 and p53 expression was seen in 31 (61%) and 39 (76%) of the tumours, with no association between either or with tumour stage or grade. There was no difference according to Bcl-2 positivity in the salvage cystectomy rate (p=0.83) or survival (p=0.68) for the 51 patients as a whole, but Bcl-2-negative patients receiving neoadjuvant cisplatin did have a significantly improved prognosis, with a median survival of 72 months compared to 17 months in Bcl-2-positive patients, and a five year survival rate of 55% (p=0.03).

**Conclusions** Bcl-2 quantification in patients undergoing radiotherapy for advanced bladder cancer identifies patients who may benefit from neoadjuvant chemotherapy. Further studies of members of the Bcl-2 family and related proteins are warranted, to define interactions between them that may contribute to oncogenesis and resistance to standard treatments. This may allow targeting of specific treatments to patients known to be sensitive to them and aid the future development of novel therapies.

### 8.4 CARBOGEN AND NICOTINAMIDE WITH RADICAL RADIOTHERAPY FOR BLADDER CANCER: UPDATED PHASE II RESULTS AND LAUNCH OF THE BCON PHASE III RANDOMISED TRIAL

PJ Hoskin*, H Phillips, S Jackson, P Wims, N Verma, MI Saunders, Mount Vernon Hospital, Northwood HA6 2RN, UK

Carbogen and nicotinamide used as radiosensitisers to overcome chronic diffusion-related hypoxia and acute perfusion-related hypoxia respectively have been evaluated in a Phase II trial in bladder cancer. A total of 102 patients have been entered at 31st January 2000 of whom 95 have completed treatment with a minimum of 6 months follow up.

Previous analyses have demonstrated an improvement in overall survival, progression-free survival and local relapse-free survival compared to historical controls. With a median follow up of 36 months this larger cohort of patients continues to maintain the previously observed improvement. The 3 year overall survival is 52%. The 3 year progression-free survival is 70% and the 3 year local relapse-free survival is 82%. No local relapse beyond 3 years have been observed. The more recent 32 patients have had a modified treatment with the concentration of carbogen gas changed to 2% $CO_2$ 98% oxygen compared to a 5% $CO_2$ 95% oxygen mixture and the dose of nicotinamide reduced from 80 mg/kg to 60 mg/kg. This was to improve compliance with the medication and has been entirely successful with all patients tolerating carbogen without difficulty throughout the 20 fraction radiotherapy schedule; 75% have taken nicotinamide with > 75% of the overall treatment (ie. > 15 fractions) and 67% for the entire treatment – this compares to only 42% taking nicotinamide 80 mg/kg throughout in the earlier patients. No significant difference is seen between these patients and the preceding cohort of 70 patients in local control, progression-free survival and overall survival curves.

Detailed toxicity analyses are underway. One patient has developed Grade III bowel toxicity and one patient has required cystectomy for a fibrosed shrunken bladder in preference to continued catheter drainage. No other major late normal tissue toxicity has been encountered.

Against the above background a formal Phase III trial of carbogen and nicotinamide in the radical treatment of bladder cancer with radiotherapy has now been launched. This uses 2% carbogen, 60 mg/kg nicotinamide with radical radiotherapy either 55 Gy in 20 fractions over 4 weeks or 64 Gy in 32 fractions over 6 1/2 weeks randomised against radiotherapy alone as the control arm. This is a multicentre study funded by the Cancer Research Campaign co-ordinated through Mount Vernon Hospital and centres are invited to collaborate.

### 8.5 FINAL ANALYSIS OF THE MRC/EORTC TRIAL OF 3 VS 4 × BEP AND 5 DAYS VS 3 DAYS PER CYCLE IN GOOD PROGNOSIS GERM CELL CANCER

JT Roberts, R de Wit, PM Wilkinson, PHM de Mulder, GM Mead, SD Fossa, PA Cook, L de Prijck, SP Stenning and L Collette, For the MRC Testicular Tumour Group, London, UK and the EORTC Genito-Urinary Group, Brussels, Belgium

This trial was designed as a 2×2 factorial equivalence trial comparing 3 cycles of bleomycin, etoposide, cisplatin (3BEP) versus 4 cycles (3BEP-1EP), and the administration of BEP over 5 days versus 3 days in IGCCCG good prognosis germ cell cancer (JCO 1997;15:594). The aim was to rule out a 5% decrease in the 2-year progression-free survival (PFS) rate with 90% power using 1-sided tests and $\alpha$=0.10. BEP consisted of etoposide 500 mg/m$^2$, administered at either 100 mg/m$^2$ days 1–5, or 165 mg/m$^2$ days 1–3, cisplatin 100 mg/m$^2$, administered at either 20 mg/m$^2$ days 1–5, or 50 mg/m$^2$ days 1–2. Bleomycin 30 mg was administered on days 1,8,15, during cycles 1–3. The randomization procedure in the MRC allowed some investigators to participate only in the comparison of 3 vs. 4 cycles. Between March 1995 and April 1998, 812 patients were randomized between 3 and 4 cycles, of these 681 were also randomized between 5 days and 3 days. Histology, marker values, and disease extent are well balanced in the treatment arms in the 2 comparisons. The median follow-up is 25 months, a minimum of 2 years follow-up is available for 93% of patients. The estimated 2 y PFS is 90.4% on 3 cycles and 89.4% on 4 cycles. The difference in PFS between 3 and 4 cycles is –0.99% (80% ci: –3.81% to +1.83%). Equivalence for 3 vs. 4 cycles is claimed as the upper bound of the 80% ci. is < 5%. In the 5 vs 3 days comparison, the estimated 2 y PFS is 88.8% and 89.7%, respectively (difference –0.92%, 80% ci: –4.1 to +2.2%). Hence, equivalence is claimed in this comparison also. Myelotoxicity was more pronounced in the 3-day schedule (test for trend leucopenia p = 0.014, thrombocytopenia p = 0.001), but this did not result in greater dose attenuations. Frequencies of non-hematological toxicities were similar. We conclude that 3 cycles of BEP, with etoposide at 500 mg/m$^2$, is sufficient therapy in good prognosis germ cell cancer, and that the administration of the chemotherapy in 3 days has no detrimental effect on the effectiveness of the BEP regimen.

### 8.6 PACLITAXEL-CONTAINING HIGH DOSE CHEMOTHERAPY FOR RELAPSED OR REFRACTORY GERM CELL TUMOURS

IA McNeish, EJ Kanfer, R Haynes, R Agarwal, SJ Harland, ES Newlands, MJ Seckl, Departments of Medical Oncology and Haematology, Imperial College School of Medicine, Hammersmith and Charing Cross Hospitals, London: The Meyerstein Institute of Oncology, Middlesex Hospital, London, UK

Previous reports from our group[1] and others[2,3] have indicated that high dose regimes containing etoposide, carboplatin and an oxazaphospharine can salvage 30–40% of patients with relapsed or refractory germ cell tumours and produce median event-free intervals of approximately 4 months. Since March 1995, we have treated 22 gonadal germ cell tumour patients with CarbopEC-T, a paclitaxel-containing high dose regime (paclitaxel 75 mg/m$^2$, etoposide 450 mg/m$^2$, carboplatin AUC10 on days –7, –5 and –3 and cyclophosphamide 60 mg/kg on days –5 and –3) followed by peripheral blood stem cell infusion (day 0). By the criteria of Beyer et al[2], 15 patients had cisplatin sensitive disease, 3 refractory disease and 4 absolutely refractory disease prior to high dose therapy. The one year overall and event-free survival rates for all patients are 68% and 51% respectively (median follow up 12.3 months). For the 15 patients with sensitive disease, these survival rates are 84% and 60% respectively, whilst for those with refractory or absolutely refractory disease, the one year overall survival is 26% with a median of 8.5 months. Six patients (response to high dose therapy: 2 CR, 4 PR) have relapsed at a median duration of 6.3 months, five of whom have subsequently died. One heavily pre-treated patient with a primary CNS germinoma has also received CarbopEC-T, and achieved PR, but relapsed at 20 months. There have also been three treatment-related deaths, all associated with pneumonitis. Pulmonary toxicity has previously been reported to complicate the addition of paclitaxel to other high dose regimes[4] but may be alleviated by administering the drug as a 120 hour infusion[5]. In summary, we believe that CarbopEC-T may enable a greater proportion of patients with relapsed and refractory germ cell tumours to enter long term remission.

1 *Br J Cancer* (1998) **77:** 1672–6
2 *J Clin Onc* (1996) **14:** 2638–45
3 *Eur J Cancer* (1998) **34:** 1883–8
4 *Blood* (1997) **90:** Supp 1 4356
5 *Cancer* (1998) **83:** 1540–5

### 8.7 MAINTENANCE TREATMENT WITH INTERFERON FOR ADVANCED OVARIAN CANCER

G Hall*[1], R Coleman[2], M Stead[3], E Gurney[3], A Phillips[3], J Brown[3], D Dassu[3], A Jenkins[1], S Brown[2], B Hancock[2], P Selby[1], T Perren[1], [1]ICRF Cancer Medicine Research Unit, St James's University Hospital, Leeds, UK; [2]Weston Park Hospital, Sheffield, UK; [3]Northern and Yorkshire Cancer Trials Research Unit, Leeds University, Leeds, UK

Patients with advanced ovarian cancer respond well to initial chemotherapy but such responses are rarely maintained, ultimately leading to the death of the patient. Interferon-alpha has been demonstrated to have activity as a maintenance therapy in patients with B-cell malignancies such as multiple myeloma. We have therefore performed a multi-centre randomised controlled trial to assess the effect of low dose subcutaneous interferon-alpha in patients with advanced epithelial ovarian cancer, in maintaining the response achieved with primary surgery and chemotherapy and its effect on overall survival.

Patients with epithelial ovarian cancer (FIGO stage Ic-IV) were registered within the trial following primary surgery/biopsy and prior to the administration of chemotherapy. The choice of chemotherapy was not dictated but the use of standard regimens including platinum agents was advised. Following chemotherapy, consenting patients with no evidence of disease progression were randomised to treatment with interferon-alpha-2a (RoferonR–Roche) at a fixed dose of 4.5 MU subcutaneously on Monday, Wednesday and Friday each week or observation. Treatment was discontinued for disease progression, toxicity or at the patient's request.

Between February 1990 and July 1997, 559 patients were registered prior to chemotherapy by 28 consultants. 300 (54%) patients were subsequently randomised; 149 to maintenance interferon, 151 to observation. Baseline characteristics of both groups including age, histology, FIGO stage at presentation and performance status were similar. 93% of patients received either single agent platinum or a platinum containing combination. A median follow-up of 26.3 months has now been achieved. 215 (71%) patients have documented disease progression and 185 (62%) have died.

For all patients randomised no significant differences exist for overall survival (median 26.9 months, interferon; 31.2 months observation; log rank p=0.56) or progression-free survival (median 11.0 months, interferon; 10.8 months observation; log rank p=0.62). Analysis of the 180 patients apparently disease-free following surgery and/or chemotherapy shows a median progression-free survival of 16.5 months (interferon) and 14.4 months (observation).

In this relatively small study, low dose subcutaneous interferon-alpha has no apparent benefit as a maintenance therapy in patients with advanced ovarian cancer.

### 8.8 MOLECULAR PROFILING OF ANGIOGENESIS AND OCCULT MICROMETASTASES IN CERVICAL CANCER

Van Trappen P, Gyselman V, Ryan A, Lowe D, Dorudi S, Shepherd J & Jacobs I, Academic Departments of Gynae-Oncology, St Bartholomew's Hospital, London (e-mail: p.o.vantrappen@mds.qmw.ac.uk)

Cervical cancers metastasize preferentially into the lymphatic system and the incidence of histologically involved lymph nodes is directly related to the stage of disease. Several prognostic factors such as microvessel density, as marker for angiogenesis, have been implicated in lymph node (LN) involvement. However, recent reports suggest that new lymphatic formation, lymphangiogenesis, might be crucial in the process of lymphatic spread. Furthermore, a subgroup of patients with node-negative early stage disease will develop recurrence despite histologically normal LNs, suggesting the presence of occult micrometastases at initial presentation.

We have therefore designed the present study to assess, by using real-time quantitative RT-PCR (Taqman) for CK 19-mRNA, the incidence and amount of occult micrometastases in 156 LNs from 32 early stage cervical cancer patients. Furthermore, we have quantified the mRNA levels of different angiogenic factors in the primary tumours.

Evidence suggestive of occult micrometastases was detected in 58% of histologically uninvolved lymph nodes from above patients. In the primary tumours a 10× increase in transcription level was observed for VEGF 121 (p<0.0000002), VEGF 165 (p<0.000002), VEGF 189 (p=0.001), thrombospondin 2 (p<0.002) and eIF4E (p<0.002). A 100× increase was observed for MMP 9 (p<0.00004) and a 1000ts increase for the lymphangiogenic factor VEGF C (p<0.000006). No significant upregulation was seen for bFGF and MMP2.

Our findings demonstrate the crucial role of VEGF A splice variants, VEGF C and MMP 9 in the process of occult metastatic spread in early stage cervical cancers.

